# Structure of Lipid Kinase p110β/p85β Elucidates an Unusual SH2-Domain-Mediated Inhibitory Mechanism

**DOI:** 10.1016/j.molcel.2011.01.026

**Published:** 2011-03-04

**Authors:** Xuxiao Zhang, Oscar Vadas, Olga Perisic, Karen E. Anderson, Jonathan Clark, Phillip T. Hawkins, Len R. Stephens, Roger L. Williams

**Affiliations:** 1 Medical Research Council Laboratory of Molecular Biology, Hills Road, Cambridge CB2 0QH, UK; 2 Inositide Laboratory, Babraham Institute, Babraham Research Campus, Cambridge CB22 3AT, UK; 3 Babraham Bioscience Technologies, Cambridge, CB22 3AT, UK

## Abstract

Phosphoinositide 3-kinases (PI3Ks) are essential for cell growth, migration, and survival. The structure of a p110β/p85β complex identifies an inhibitory function for the C-terminal SH2 domain (cSH2) of the p85 regulatory subunit. Mutagenesis of a cSH2 contact residue activates downstream signaling in cells. This inhibitory contact ties up the C-terminal region of the p110β catalytic subunit, which is essential for lipid kinase activity. In vitro, p110β basal activity is tightly restrained by contacts with three p85 domains: the cSH2, nSH2, and iSH2. RTK phosphopeptides relieve inhibition by nSH2 and cSH2 using completely different mechanisms. The binding site for the RTK's pYXXM motif is exposed on the cSH2, requiring an extended RTK motif to reach and disrupt the inhibitory contact with p110β. This contrasts with the nSH2 where the pY-binding site itself forms the inhibitory contact. This establishes an unusual mechanism by which p85 SH2 domains contribute to RTK signaling specificities.

## Graphical abstract

**Graphical Abstract:**

**Figure:**
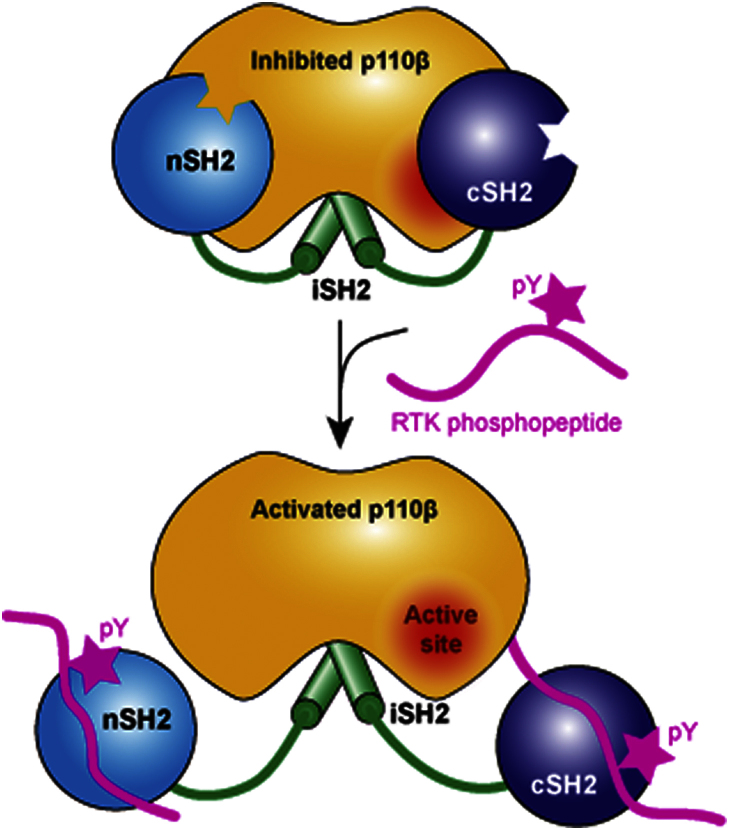

**Highlights:**

► Both nSH2 and cSH2 domains of p85 inhibit basal activity of p110β ► p110β/p85β structure shows cSH2 contacts the C terminus of p110β ► Relief of cSH2 inhibition, unlike nSH2, requires extending beyond the pYXXM motif ► p110β C terminus is critical for phosphorylation of lipids and activation by RTKs

## Introduction

The class IA PI3Ks are obligate heterodimers, consisting of a p110 catalytic subunit and a p85-type regulatory subunit. They trigger a cascade of mammalian signaling pathways downstream of receptor tyrosine kinases (RTKs) by generating the lipid second messenger phosphatidylinositol 3,4,5-trisphosphate (PIP_3_), which recruits effectors to lipid membranes (Engelman et al., 2006; Vanhaesebroeck et al., 2010). Among PI3Ks, the p110β isotype is uniquely activated by both RTKs and Gβγ heterodimers (Kurosu et al., 1997; Maier et al., 1999; Guillermet-Guibert et al., 2008). p110β controls platelet activation and thrombosis (Jackson et al., 2005; Martin et al., 2010), DNA replication (Marqués et al., 2009), and male fertility (Ciraolo et al., 2010) and contributes to insulin signaling (Ciraolo et al., 2008; Knight et al., 2006). Moreover, p110β has oncogenic functions in PTEN-negative tumors and in ERBB2-dependent tumors (Jia et al., 2008; Ciraolo et al., 2008).

**Figure 1:**
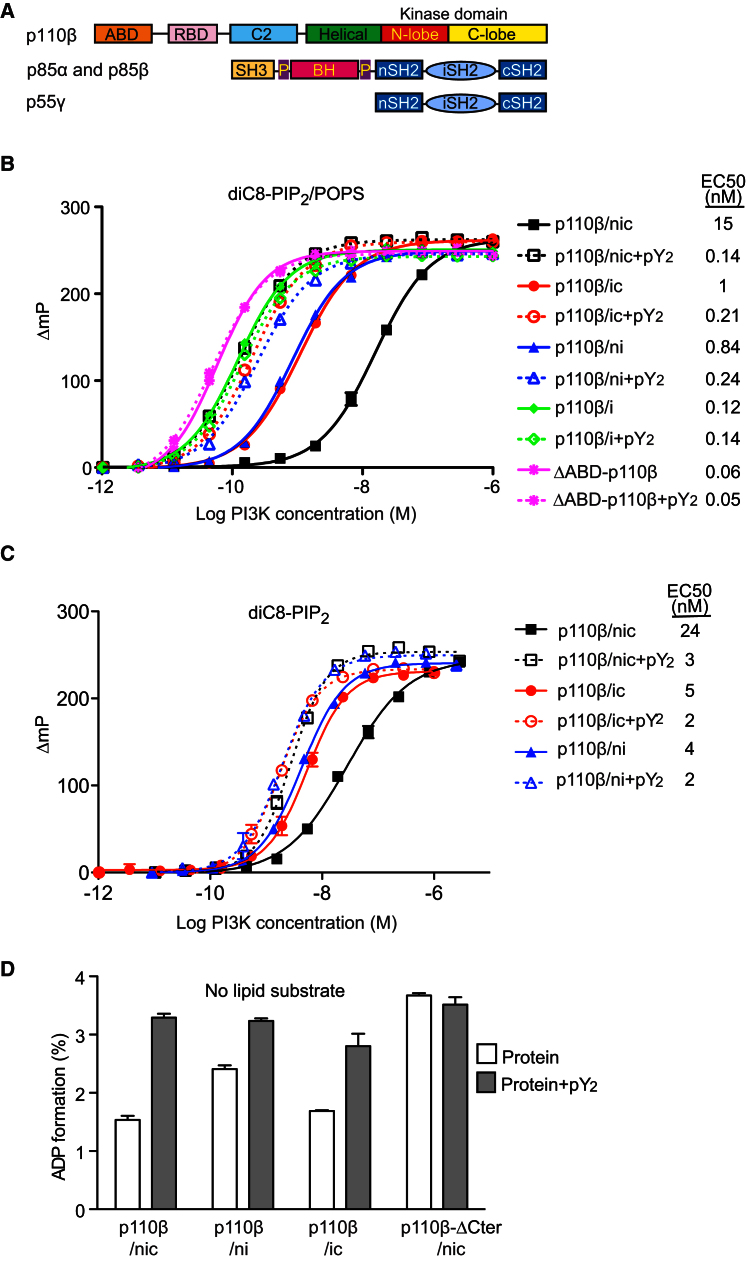
Inhibition of p110β by p85-Type Regulatory Subunits and Activation of the Complexes by RTK Phosphopeptide (A) Domain organization of p110β and the regulatory subunits p85α, p85β, and p55γ. The color scheme for the domains is used for all figures, unless otherwise stated. (B) Kinase activity with diC8-PIP_2_/POPS liposomes as a function of enzyme concentration (measured by ADP formation) shows the inhibitory effects of p85β-nicSH2 (nic) and p85β-icSH2 (ic) on the basal activity of p110β. The inhibition is released upon addition of the 10 μM PDGFR pY_2_. The free catalytic subunit (ΔABD-p110β) is more active than any complex. The y axis is expressed as a change in fluorescence polarization (ΔmP), which is obtained by subtracting the observed polarization for the construct at a given enzyme concentration from the maximum fluorescence polarization for that construct. The plateau in these assays arises due to the competitive nature of the ADP detection system, based on displacement of ADP-Alexa 633 tracer from the ADP^2^-antibody by ADP generated during the PI3K assay (see Supplemental Experimental Procedures). All measurements were done in triplicates, and the error bars indicate the standard error of the mean (SEM). (C) Kinase activity of p110β/p85β complexes with monomeric substrate (75 μM diC8-PIP_2_) in the presence and absence of pY_2_; y axis as in (B). (D) Basal- and pY_2_-stimulated activities of 35 nM PI3K complexes in the absence of lipids (ATP hydrolysis). Activities are given as percent of ATP converted to ADP using the Transcreener assay. Bars indicate SEM.

All class IA PI3Ks can induce oncogenic transformation (Zhao et al., 2005; Kang et al., 2006). Consequently, p110α, p110β, and p110δ need to be tightly regulated. This is achieved by the regulatory subunits, which stabilize the p110, inhibit its basal activity, and facilitate recruitment and activation downstream of RTKs. There are five p85-type regulatory subunits (p85α, p85β, p55γ, p55α, and p50α) encoded by three genes. All regulatory subunits have a coiled-coil inter-SH2 domain (iSH2) that binds tightly to the adaptor-binding domain (ABD) of the p110 catalytic subunit (Figures 1A and S1). The iSH2 is flanked by the two SH2 domains, nSH2 and cSH2. Upon RTK activation, the p85 SH2 domains bind to a phosphotyrosine (pY)-containing consensus sequence, pYXXM, in RTKs and pY-phosphorylated adaptor proteins (Carpenter et al., 1993; Songyang et al., 1993). Although, in general, short pY motifs from RTKs provide tight binding to SH2 domains (Schlessinger and Lemmon, 2003; Pawson and Nash, 2003; Huang et al., 2008), the recent structure of the phospholipase Cγ (PLCγ) SH2 domains with the activated tyrosine kinase domain of FGFR1 has elegantly challenged the notion that short, linear polypeptides can recapitulate the SH2 interaction with their native targets (Bae et al., 2009).

Mutations in the p85α regulatory subunit are frequent in several cancers and promote cell survival and oncogenesis in a p110-dependent manner (Parsons et al., 2008; Cancer Genome Atlas Research Network, 2008; Jaiswal et al., 2009). Binding SH2 domains of a p85 regulatory subunit to RTKs relieves the basal inhibition and leads to activation of the p110 catalytic subunit (Backer et al., 1992; Herbst et al., 1994). The minimal regulatory subunit capable of inhibiting p110α consists of the nSH2 and iSH2 (p85-niSH2) (Yu et al., 1998). The role of the cSH2 has remained elusive, since it is required for full activation of p110α in the context of the full-length p85 regulatory subunit (Rordorf-Nikolic et al., 1995), but it has no role in inhibiting basal activity of p110α (Yu et al., 1998). Several cSH2 truncated versions of p85α have been isolated from cancer cell lines, including p65 (lacking the cSH2 and part of the iSH2) and p76 (lacking the C-terminal 85 residues of the cSH2) (Jimenez et al., 1998; Jücker et al., 2002). Both truncations are associated with increased PI3K activity, contributing to cellular transformation.

Intrigued by the oncogenic potential of all p110 isoforms, we tried to uncover the critical inhibitory mechanisms that keep these enzymes under control. We found that the cSH2, in addition to nSH2, has a key role in p110β inhibition. To understand the basis for the unanticipated inhibition by the cSH2, we determined the crystal structure of the p110β catalytic subunit in a complex with a regulatory construct consisting of the iSH2 and cSH2 p85-icSH2. The structure of this complex shows that the cSH2 interacts with a “regulatory arm” at the C terminus of the kinase domain. This contact inhibits the enzyme and the inhibition can be relieved both in vitro and in cells by RTK phosphopeptides or by a point mutation in the cSH2. The cSH2 surface that makes the inhibitory contact with p110β does not coincide with the pY-binding site, providing a structural basis for the requirement of extended pYXXM RTK motifs to relieve p110β inhibition.

## Results

### Inhibition of p110β by p85β nSH2 and cSH2 Domains

Given the substantial evidence for an oncogenic potential of p110β (reviewed in Vogt et al., 2009), we focused on the regulation of this isoform. We assayed the ability of a series of p85 deletion variants to inhibit full-length p110β as well as the extent to which this inhibition is relieved by RTK phosphopeptides. By using several types of substrates—liposomes, soluble lipids, and water—we tried to deconvolute regulation arising from effects on membrane binding, monomeric lipid interaction, and the intrinsic chemistry of the phosphoryl transfer.

We compared the catalytic activity of purified recombinant proteins using either a high-throughput fluorescence polarization assay, which measures the production of ADP (Klink et al., 2008) (Supplemental Experimental Procedures), or assays that measure PIP_3_ production directly (using radioactive assays and mass spectroscopy). The activity of the p110β catalytic subunit with the lipid substrate diC8-PIP_2_/POPS is strongly inhibited by a p50-like regulatory subunit core that contains both the nSH2 and cSH2, linked through the iSH2 (nicSH2) (Figure 1B). The basal activity of the p110β/p85β-nicSH2 is considerably lower than a complex containing only the iSH2 or a construct of the catalytic subunit without the regulatory subunit, ΔABD-p110β. We used ΔABD-p110β as a substitute for the free full-length catalytic subunit, since the latter cannot be stably expressed in insect cells (Berndt et al., 2010).

To dissect the regulatory roles of each of the three domains present in the p85β-nicSH2 construct, we assayed further truncation variants of p85β for their ability to inhibit the basal activity of p110β. Our results clearly show that both nSH2 and cSH2 contribute significantly to inhibition of p110β. Each SH2 domain inhibits the enzyme relative to the complex containing only iSH2 (Figure 1B). Compared to the complex with both SH2 domains (nicSH2), the absence of either the nSH2 or the cSH2 leads to enzyme activation as measured by ADP formation (Figure 1B) or by PIP_3_ formation using radioactive assays or mass spectroscopy (Figures S2A–S2C and S3A). This surprising inhibitory effect of the cSH2 on the p110β basal activity stands in contrast to p110α, which was reported previously to be inhibited by the nSH2 but not by the cSH2 (Yu et al., 1998).

### Activation by RTK Bis-Phosphorylated Peptide

The addition of a bis-phosphopeptide (PDGFR 735–767, with pY740 and pY751, abbreviated as pY_2_) stimulates kinase activity (measured by ADP formation) by all three complexes containing SH2 domains (nicSH2, niSH2, or icSH2), but did not affect the activity of p110β/p85β-iSH2 or ΔABD-p110β (Figure 1B). Similarly, pY_2_ stimulates lipid kinase activity of p110β SH2-containing complexes, as measured directly by PIP_3_ formation (Figures S2A–S2C) using a radioactive phosphorylation assay (Knight et al., 2007). We find that the fold of activation by pY_2_ depends greatly on the type of lipid substrate and lipid presentation (10-fold activation with a defined mixture of “brain lipids” [composed of 5% PIP_2_, 20% PS, 15% PC, 45% PE, 5% sphingomyelin, and 10% cholesterol] and 16-fold with diC8-PIP_2_/POPS liposomes, measured by PIP_3_ formation for p110β/p85β-nicSH2 [Figures S2A–S2C]). The variation in the activation fold is not surprising given the long history of reports on the huge effect of the lipid composition and vesicle size on the basal activity of PI3Ks (e.g., micelle/vesicle composition, vesicle size, PI versus PIP_2_, etc.) (Hübner et al., 1998; Chaussade et al., 2009; Carpenter et al., 1990; Beeton et al., 2000). Previously reported activation by phosphopeptides range from 2- to 4-fold for immunoprecipitated p110α/p85α, using pure PI substrate (Yu et al., 1998; Miled et al., 2007), to 20- to 40-fold for purified recombinant p110α/p85α, p110β/p85α, and p110δ/p85α using PIP_2_-containing liposomes (Maier et al., 1999).

p110β complexes can also phosphorylate a soluble lipid substrate diC8-PIP_2_, although less efficiently than liposomal substrate. Interestingly, phosphorylation of monomeric diC8-PIP_2_ is still activated by pY_2_, with a change in EC_50_ upon peptide addition ranging from 2- to 8-fold, depending on the regulatory construct (Figure 1C).

All PI3Ks have ATPase activity in addition to their lipid kinase activity (Miller et al., 2010; Klink et al., 2008) that can be observed when measuring ADP formation in the absence of lipid substrate (Figure 1D); however, the ATPase activity is quite low compared with the lipid kinase activity. We have verified that this ATPase activity is not due to a contaminating ATPase, because it can be fully inhibited by a kinase-dead mutation (D913N) in the recombinant enzyme or by a PI3K-specific inhibitor (Figure S3B). This ADP-formation assay has enabled us to expand our insight into mechanisms of PI3K regulation by determining activity both in the presence and absence of lipid substrates.

### Structural Basis for p110β Inhibition by cSH2 and Release of Inhibition by Phosphopeptide

In an attempt to unravel the structural basis for the inhibition of p110β by the cSH2, we crystallized full-length mouse p110β in a complex with the mouse p85β-icSH2 (residues 423–722) in the presence of the PI3K inhibitor GDC-0941. The structure was refined at 3.3 Å resolution. There is one copy of a 1:1 p110β/p85β complex in the asymmetric unit, and the density for GDC-0941 is clearly visible (see Table S1 for contacts). Crystallographic statistics are given in Table 1.

**Figure 2:**
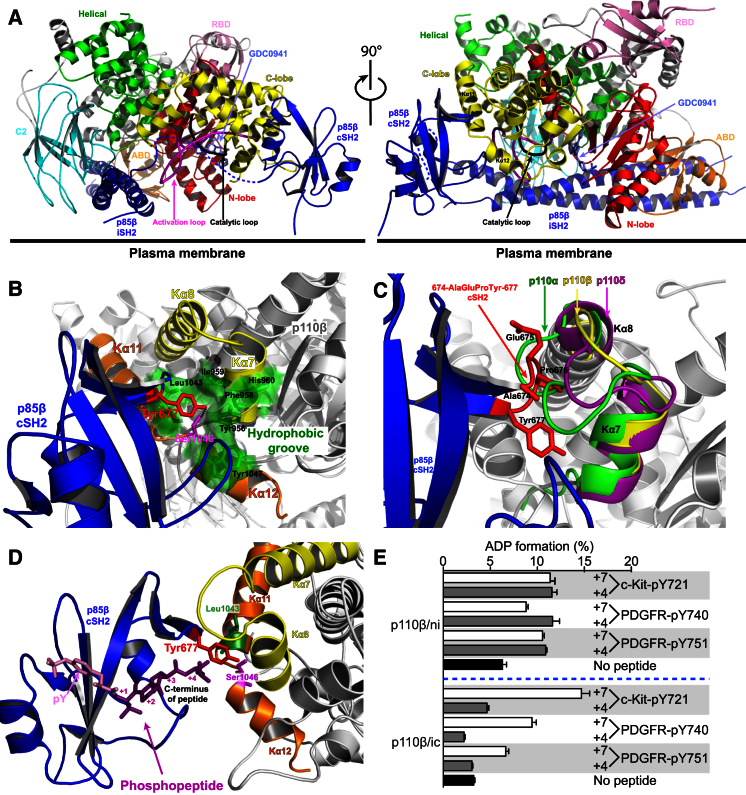
Structure of p110β in Complex with p85β-icSH2 (A) Cartoon representation of the p110β/p85β-icSH2 complex with GDC0941 (light blue sticks). Views from the front and side (turned 90°) are shown. The ordered activation loop is colored in magenta and the catalytic loop in black. (B) Detailed view of the contacts between the p85β-cSH2 and the C-lobe of the kinase domain. The main contact residue Tyr677-p85β (red) interacts with Ser1046-p110β (magenta) and the hydrophobic groove (pale green) formed by two “arms,” Kα7/Kα8 (yellow) and Kα11/Kα12 (orange). Dashed black line is a potential hydrogen bond between Tyr677-p85β and Ser1046-p110β. (C) Cartoon of the p110β Kα7/Kα8 “arm” (yellow) superimposed on p110α (green) and p110δ (magenta), suggesting a steric clash between cSH2 protrusion (674-AlaGluPro-676) (red stick) and Kα7/Kα8 loop of p110α. (D) Cartoon of the cSH2 binding to RTK phosphopeptide, modeled as in the crystal structure of free cSH2 in complex with a PDGFR phosphopeptide (magenta; PDB ID: 1H9O). (E) Effects of different RTK phosphopeptides (10 μM) on activity of niSH2 and icSH2 complexes with p110β (1 nM), showing selective requirement for peptides longer than pY + 4 for disinhibition of the cSH2. Error bars indicate SEM.

This complex shows that both the iSH2 and cSH2 are ordered and interact with the p110β catalytic subunit. In the overall arrangement of the two subunits, the long coiled coil of the iSH2 slots into the large arch formed by the catalytic subunit, while the cSH2 nestles against the C-terminal region of the kinase domain (Figure 2A). The structure of the complex reveals possible underlying mechanisms of inhibition by the cSH2. In contrast to the nSH2 of p85α, which is in contact with three domains of p110α (C2, helical, and kinase domains) (Mandelker et al., 2009; Miled et al., 2007), the cSH2 of p85β contacts only the C lobe of the p110β kinase domain. The solvent accessible surface area buried by the cSH2/p110β interface (1264 Å^2^) is smaller than the nSH2/p110α interface (1793 Å^2^). Our structure shows that a cSH2 loop (Ala674 to Tyr680), which was described as a protrusion at the surface of cSH2 (Hoedemaeker et al., 1999), forms the main contact point with the p110β. This loop interacts with a double layer of helical pairs, the Kα7/Kα8 and the C-terminal Kα11/Kα12 of the kinase domain (Figures 2B and S2D). We will refer to each of these helical pairs as an “arm” and the loop between the two helices as an “elbow.” The Kα11/Kα12 elbow is marked by Ser1046, which is at the center of a hydrophobic groove between the two elbows, including residues Tyr956, Ile959, and Leu1043. This hydrophobic groove accommodates Tyr677 in βF of the cSH2 (SH2 secondary structure nomenclature follows the description in Eck et al., 1993), and Ser1046-p110β is positioned to form a potential hydrogen bond to the side chain of Tyr677-p85β. Curiously, this conserved tyrosine in the cSH2 is an insertion relative to the nSH2 (Hoedemaeker et al., 1999). This unique feature suggests distinct regulatory mechanisms by the nSH2 and cSH2. Other contacts between the cSH2 and the catalytic subunit, such as the potential hydrogen bond between the side chain of Glu675 in the cSH2 and the backbone of Asn969 in the Kα7/Kα8 elbow in p110β, may also contribute to the binding.

A comparison of all class IA PI3K structures suggests this inhibitory mechanism by cSH2 is likely to be important for both p110β and p110δ. Interestingly, in p110α, the Kα7/Kα8 elbow is two residues longer relative to p110β and p110δ. Therefore, the p110α elbow might clash with residues from the cSH2-p85β protrusion (674-AlaGluPro-676) (Figure 2C), contributing to the reported lack of inhibition of p110α by the cSH2. Consistent with this, when we replaced the Kα7/Kα8 elbow of p110β with the elbow of p110α, we found that the mutant is about 2-fold more active than the wild-type p110β (Figure S3C).

There is a notable difference between the ways in which the nSH2 and cSH2 of p85 bind the p110 catalytic subunit. The pY-binding site on the nSH2 is buried in the contact with the helical domain (residues 542–546 in p110α), suggesting immediately how phosphopeptide binding releases nSH2 inhibitory effects on p110α (Mandelker et al., 2009). In contrast, the pY-binding site on the cSH2 is exposed and not buried in the interface with the p110β (Figure 2D). Superposition of the cSH2 bound to a PDGFR phosphopeptide (PDB ID: 1H9O) (Pauptit et al., 2001) suggests that a peptide should have more than four residues following the pY in order to break the cSH2/p110β contact. Indeed, we found that three different RTK phosphopeptides with seven residues following the pY (pY + 7) activated the p110β/p85β-icSH2 complex, whereas pY + 4 peptides failed to activate this complex (Figure 2E). Nevertheless, all six peptides activated the p110β/p85β-niSH2 complex to a similar level. These results suggest that the fundamental differences between regulation by p85 nSH2 and cSH2 may confer much greater contextual specificity of PI3K activation by RTKs than had been anticipated. Further mapping of PDGFR-derived phosphopeptides shows that inhibition of p110β/p85β-icSH2 is relieved by pY + 5 or longer phosphopeptides (Figure S3D). In addition, relief of inhibition by pY + 5 peptides is dependent on the residue identity at the +5 position (Figure S3D). The longer phosphopeptides (pY + 5 to pY + 7) are more efficient at disinhibiting the enzyme, despite the fact that they have lower affinity for the p110β/p85β complex compared to the short phosphopeptide (pY + 4) (Figure S3E). This is consistent with the longer peptides having to compete with p110β for binding to the cSH2, whereas the pY + 4 peptide binds to the cSH2 without displacing the p110β.

### Effect of Contact Mutation on Basal Activity and RTK Activation

**Figure 3:**
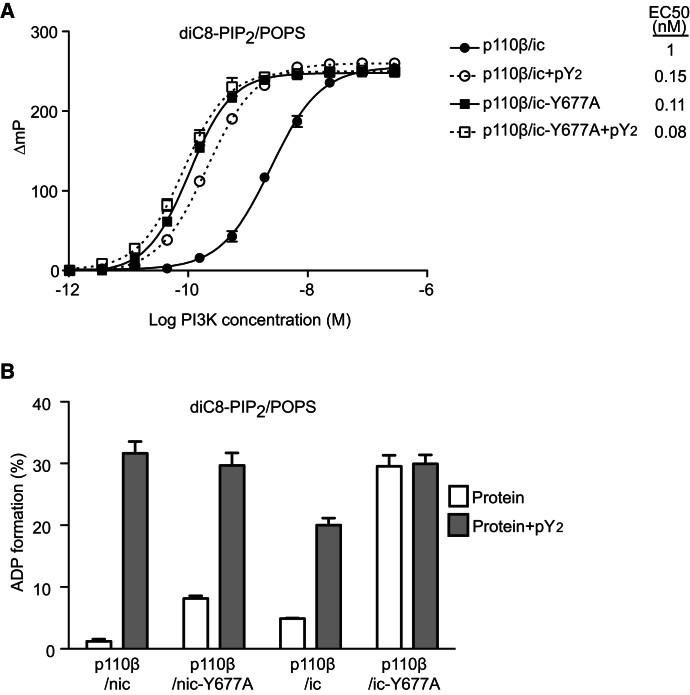
Mutation Y677A in p85β-cSH2 Releases Inhibitory Effect on p110β (A) Kinase activity (ADP formation) of p110β in a complex with p85β-icSH2-Y677A (Y677A-ic) compared to the wild-type complex (ic) in the absence and presence of 10 μM PDGFR pY_2_ (ADP formation on the y axis expressed as in Figure 1B). (B) Comparison of p110β activities of 1 nM complexes with wild-type or Y677 mutant nicSH2 or icSH2 (activity shown as in Figure 1D).

The structure of the PI3Kβ complex has identified Tyr677 in the p85β cSH2 as the major contact with the elbow region of the p110β. We mutated Tyr677 to alanine to see if this contact regulates p110β activity. The mutant p110β/p85β-icSH2-Y677A complex in the absence of pY_2_ is more active than the wild-type p110β/p85β-icSH2, and it is not activated by pY_2_ (Figure 3A). Similarly, the p110β complex with nicSH2-Y677A, a regulatory construct containing wild-type nSH2 but mutant cSH2-Y677A, has a basal activity higher than the wild-type (Figure 3B), demonstrating that the inhibitory grip of the cSH2 on p110β can be released by a single mutation in the cSH2. Mutation of another residue, E675A, from the AlaGluPro sequence preceding Tyr677, partially relieves cSH2-mediated inhibition (Figure S3F), consistent with this residue forming part of the interface with the p110β subunit. Alanine from the AlaGluPro sequence in p85α was found mutated in a colorectal cancer (A682V in p85α) (Jaiswal et al., 2009). This mutation slightly activates p110β (Figure S3F).

Mutation L1043H in the hydrophobic patch on the surface of p110β that accommodates Tyr677 also increased basal activity, consistent with diminished grip of the cSH2 (Figure S3G). Interestingly, the p110β-L1043H mutant, which is the reverse of the oncogenic H1047L in p110α, has a lower activity than wild-type p110β in the presence of pY_2_.

### All Three Types of Regulatory Subunits Modulate p110β Activity via the Same Contact

**Figure 4:**
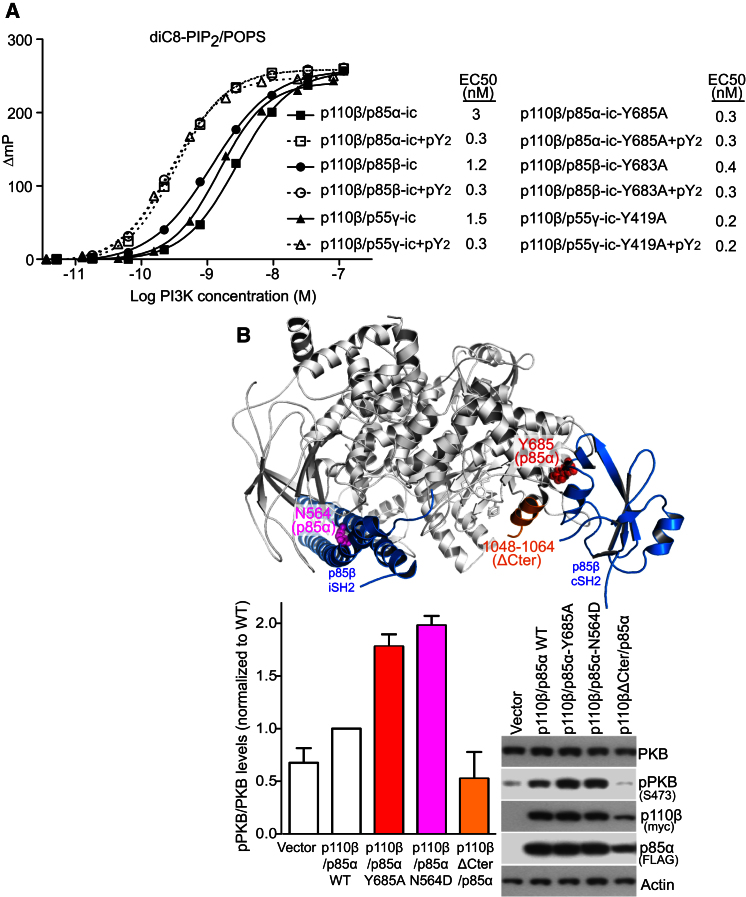
cSH2 Is Important for p110β Inhibition In Vitro and in Cells (A) Kinase activity of human p110β and icSH2 wild-type constructs from three human regulatory subunits (p85α, p85β, and p55γ) in the absence and presence of 10 μM PDGFR pY_2_ (y axis as in Figure 1B). EC_50_s for wild-type and icSH2 tyrosine mutants, in the absence and presence of 10 μM PDGF pY_2_, are shown. (B) Western blots of HEK cells transiently expressing wild-type and mutant human p110β/p85α. The membrane was probed with antibodies against PKB, pPKB (pSer473), myc (p110β), FLAG (p85α), and actin. Bar graphs show mean ± SEM (n = 3) of PKB phosphorylation level normalized to wild-type p110β/p85α (WT). The position of the three mutations is mapped on the p110β/p85β structure.

Given the differential abundance and tissue distribution of the p85-related regulatory subunits, we examined whether three human regulatory subunits, p85α, p85β, and p55γ, could downregulate human p110β via their cSH2 domains. We found that pY_2_ significantly stimulated the activity of p110β bound to the icSH2 from any of the three regulatory subunits (Figure 4A). Mutation of the conserved tyrosine in p85α, p85β, and p55γ, equivalent to Tyr677 in Mmp85β, released the inhibition of p110β for all three complexes (Figure 4A). Moreover, these tyrosine-mutated icSH2 heterodimers were not significantly activated by pY_2_. This suggests that tyrosine in βF1 of the cSH2 represents the major point of contact to the p110β catalytic subunit for all three regulatory subunits. Therefore, all regulatory subunits could potentially modulate the activity of the ubiquitously expressed p110β.

### Mutation of a Contact Residue in p85α Increases Downstream Signaling in Cells

Protein kinase B (PKB or Akt) activation is directly related to the activity of class I PI3Ks, via PIP_3_, which promotes phosphorylation of PKB on Thr308 and Ser473 (Vanhaesebroeck and Alessi, 2000). Cotransfection of HEK293T cells with human wild-type p110β and p85α-Y685A mutant resulted in a 2-fold increase in PKB phosphorylation relative to the wild-type heterodimer (Figures 4B and S4A). A p110β/p85α-N564D heterodimer, carrying a somatic mutation previously demonstrated to potently activate the PI3K signaling pathway (Jaiswal et al., 2009), also showed an increase in phosphorylation of PKB Ser473 (Figure 4B) and Thr308 (Figure S4A). We also quantified the amount of PIP_3_ in cells to have a direct measure of PI3K activity. Our data are consistent with both mutants increasing PI3K downstream signaling (Figure S4B).

### Effect of Kinase Domain C-Terminal Truncation on Activity

**Figure 5:**
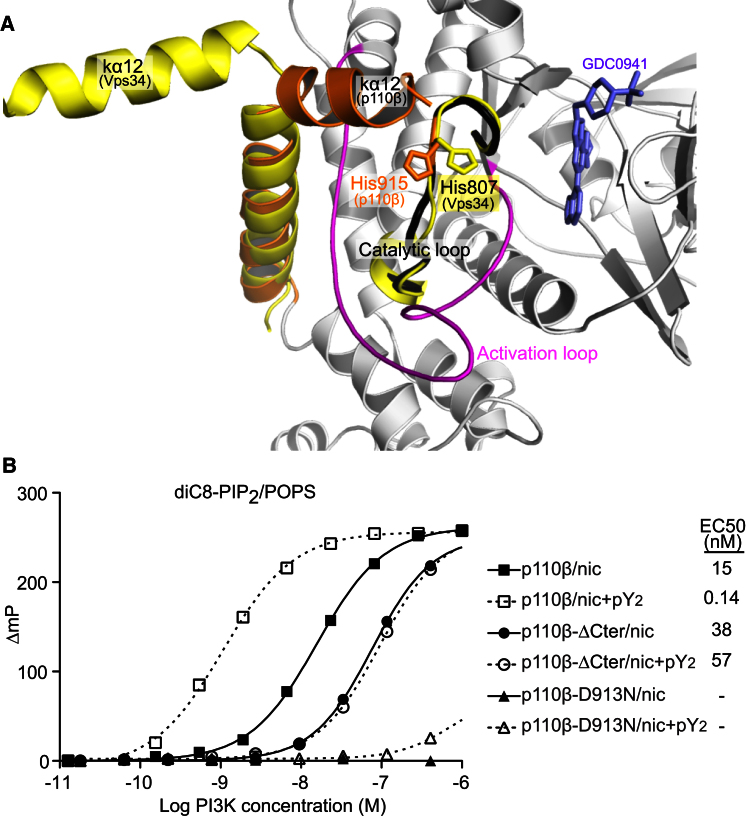
Truncation of the C Terminus in p110β Decreases Its Activity for Lipid Substrates and Increases ATP Hydrolysis in the Absence of Lipids (A) The p110β kinase domain has the signatures of an inactive conformation: C-terminal Kα12 helix folds over the activation loop and His915 (from the DRH motif) points away from the active site (orange). They differ from the same elements in the presumably active conformation of Vps34 (yellow) (PDB: 2X6H). (B) Lipid kinase activity (ADP formation) of the wild-type p110β/p85α, in comparison with a truncation mutant lacking 17 residues from the p110β C terminus (ΔCter) and a kinase-dead mutant (D913N). Activities were determined in the absence and presence of 10 μM PDGFR pY_2_. For the D913N mutant, the EC_50_ was too high to be determined accurately (y axis as in Figure 1B).

The cSH2 is in contact with C-terminal helix Kα12, suggesting that Kα12 could be implicated in the regulation of p110 by the cSH2. In the class III PI3K Vps34, the C-terminal helix (Kα12) plays a critical role in catalysis (Miller et al., 2010). The Vps34 structure seems to be in an active conformation, with the C-terminal helix pointing away from the catalytic domain and His807 (from the DRH motif) pointing toward the active site (Figure 5A). In our PI3Kβ structure, the C-terminal helix folds over the catalytic loop, presumably locking it in a closed, inactive conformation, and His915 from the DRH motif points away from the active site.

To test the importance of Kα12 in p110β, we made a truncation lacking the C-terminal 17 residues, p110β-ΔCter. This deletion mutant had very low basal activity with diC8-PIP_2_/POPS (Figure 5B) and no measurable activity with the “brain lipids” (Figure S3A). In cells, this mutant showed PKB phosphorylation levels similar to the vector control (Figure 4B). Therefore, the C terminus is important for p110β activity both in vitro and in cells. Interestingly, the ATPase activity in the absence of lipid substrate was increased for the C-terminally truncated p110β (Figure 1D). In Vps34, we have shown that the analogous C-terminal helix has dual roles: promoting membrane binding and lipid substrate interaction, while suppressing off-pathway activity in the absence of lipid substrate (Miller et al., 2010). Our data suggest a similar role for the C-terminal helix in p110β. By clamping this C terminus, the cSH2 likely inhibits the enzyme by preventing both productive lipid binding and phosphoryl transfer.

### Structural Basis for p110β Regulation by iSH2 of p85β

**Figure 6:**
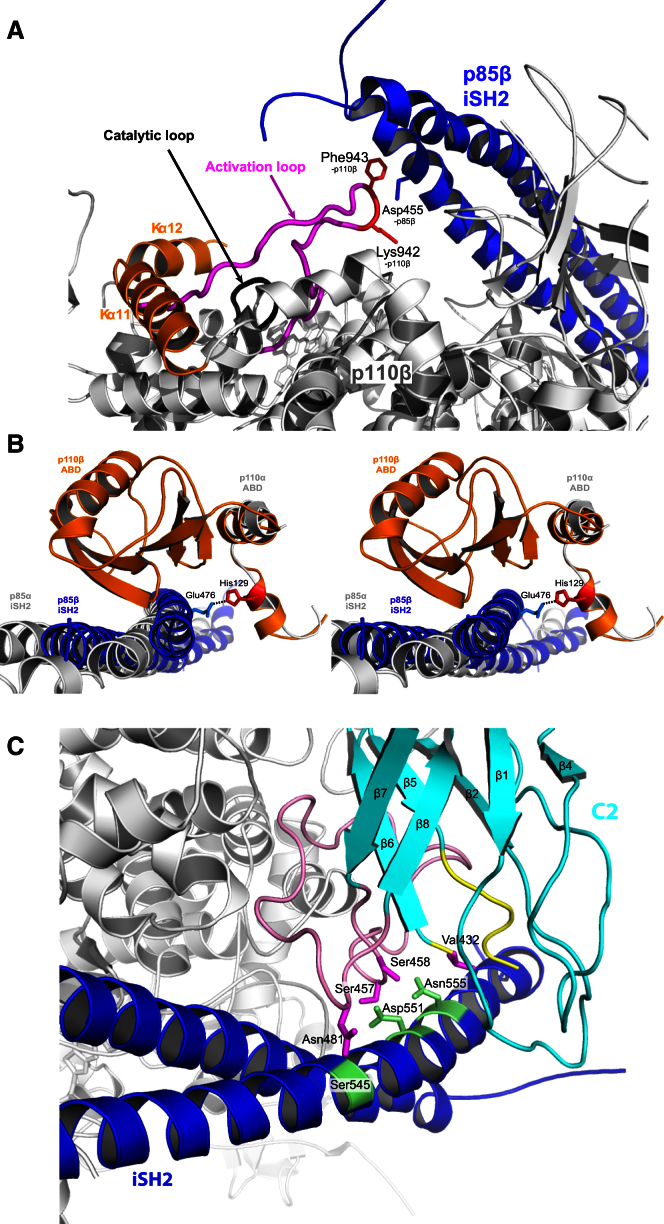
Structural Basis for p110β Regulation by p85β-iSH2 Domain (A) Close-up of interactions between p85β-iSH2 (blue) and p110β activation loop. (B) Stereo view of the unique interaction between the iSH2 (blue) with the ABD-RBD linker of p110β (orange), as compared to p110α (white). (C) Detailed interactions between residues in the iSH2 (green sticks) with residues in the C2 domain of p110β (magenta sticks). The CBR3 (Cβ5/Cβ6 loop) and Cβ7/Cβ8 loops that contact the iSH2 are highlighted in yellow and pink, respectively.

The iSH2 forms important inhibitory contacts with the p110α catalytic subunit (Wu et al., 2009). Compared to the other class I PI3Ks, the p110β catalytic subunit exhibits an ordered activation loop (residues 930–955), although the side-chain density is poor. This loop is responsible for substrate specificity (Bondeva et al., 1998). Our structure shows that the long activation loop extends all the way to the iSH2, resulting in the close vicinity of Asp455 in the iSH2-p85β (Asp464-p85α) with both Phe943 and Lys942-p110β (Figure 6A). This interaction could potentially restrict the flexibility of the activation loop and thereby contribute to inhibiting the enzyme. Mutation D464H-p85α, which was found in glioblastoma (Parsons et al., 2008), could affect interaction with Phe943 and Lys942, thereby releasing the grip of iSH2 on the activation loop.

In addition to the activation loop, p85-iSH2 contacts the ABD and C2 domains of p110β differently from the p110α/p85α complex (Figures 6B and 6C). Previous results for p110α have shown unambiguously that the iSH2/C2 contact is inhibitory (Wu et al., 2009). Two of the contact areas involve iSH2 residues that are mutated in p85α subunit in glioblastomas and were shown to activate p110α, p110β, and p110δ in vitro and in vivo (Parsons et al., 2008; Jaiswal et al., 2009; Wu et al., 2009). The Asp551-p85β, equivalent to Asp560-p85α (mutated to tyrosine in cancer), interacts with the Ser457 and Ser458 in the Cβ7/Cβ8 loop from the C2 domain and possibly also with the poorly ordered CBR1 loop (Figure 6C). D560Y mutation could disrupt the interactions this residue makes with the C2 domain. Asn555-p85β, equivalent to Asn564-p85α (mutated to lysine or aspartic acid in cancers), interacts with the largely disordered CBR3 (Cβ5/Cβ6 loop) (Figure 6C). CBR3 is rich in basic residues that are important for PI3K activity in vivo, probably through their interaction with the phospholipid membranes (Denley et al., 2008). The iSH2 contact with CBR3 may affect the membrane interaction.

The ABD-p110β retains the extensive contacts with iSH2-p85β seen previously in p110α/p85α-iSH2, resulting in 2269 Å solvent-accessible surface area being buried at the interface (Figure 6B). Among the residues in contact is Asn518-p85β, which is conserved in all regulatory subunits. The equivalent residue in p85α, Asn527, was found mutated to lysine in colorectal cancer (Jaiswal et al., 2009). The structure suggests mutation to lysine would result in a steric clash with the backbone of the Aβ1/Aβ2 loop, thus possibly affecting the primary interaction between ABD and iSH2. A major difference from p110α/iSH2 is seen in the ABD-RBD linker, where the Lα1 and Lα1′-p110β, in particular His129-p110β, are much closer to the Glu476-iSH2 than the corresponding residues in p110α (Figure 6B). Such differences could contribute to subtle variations in binding of iSH2 to distinct p110 subunits.

## Discussion

**Figure 7:**
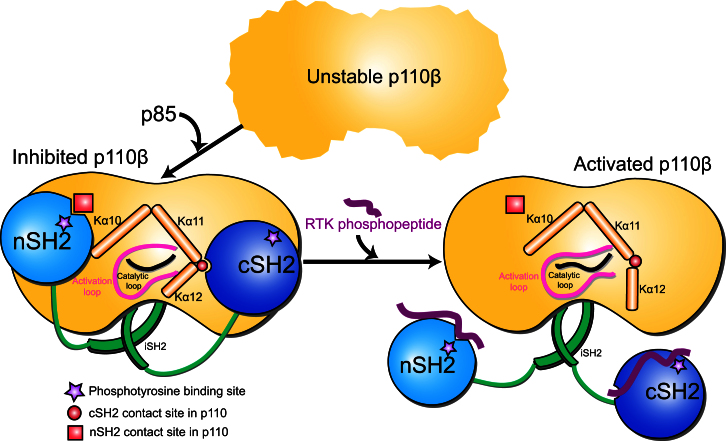
A “Three-Brake” Regulatory Model for Inhibition of p110β Basal Activity Model of the “three-brake” inhibition of p110β activity by the nSH2, iSH2, and cSH2 domains of p85. All three domains make inhibitory interactions with the catalytic subunit. The nSH2 and cSH2 are in contact with the “regulatory square” formed by the three C-terminal helices of p110β (Kα10–Kα12). This “regulatory square” encloses the catalytic and activation loop and could mediate the SH2 inhibitory effects. RTK phosphopeptide binding to SH2 domains relieves the inhibition. The main contact between nSH2 and catalytic subunit overlaps perfectly with pY-binding site on nSH2, but not on cSH2. Therefore, relief from cSH2 inhibition requires an extended pYXXM motif.

We propose three extrinsic “brakes” on the basal activity of p110β exerted by the nSH2, cSH2, and iSH2 domains of the regulatory subunit (Figure 7 and Movie S1). The nSH2 interacts with the C2, helical, and the kinase domains of p110 and provides one brake. The second brake is the contact of the cSH2 with the C lobe of the kinase domain. This inhibitory cSH2 interaction distinguishes p110β from p110α (Yu et al., 1998), and our structure suggests a possible explanation for this difference. The third brake is provided by the iSH2, which nestles under the arch formed by the catalytic subunit and forms inhibitory contacts with the C2 domain (Wu et al., 2009). Its mutations in several types of tumors lead to dramatic upregulation of all class IA isozymes. While p110β activity is inhibited by the engagement of three brakes, p110α appears to have only two brakes (nSH2 and iSH2), suggesting that p110α is poised to have greater activity when one of the brakes is lost by mutation. The inhibition of p110β by both SH2 domains of the p85 subunit may explain observations that p110β is less responsive to RTK stimulation in some cells (Kurosu et al., 1997; Maier et al., 1999; Guillermet-Guibert et al., 2008).

The inhibitory effect of the cSH2 could be explained by its contacts with the C-terminal region of the kinase domain. In our crystal structure, the kinase domain of the catalytic subunit exhibits the signatures of an inactive conformation. The contacts of the cSH2 with the regulatory elements surrounding the activation and catalytic loops, i.e., helices Kα11/Kα12 and Kα7/Kα8 forming the double-layer regulatory arm, could possibly pin the catalytic and activation loops into an inactive conformation and prevent the C-terminal helix from swinging out to its active conformation. By affecting the C-terminal helix conformation, the cSH2 may also affect membrane binding. In the primordial class III PI3K Vps34, the equivalent C-terminal helix interacts with membranes and its deletion abolishes enzyme activity (Miller et al., 2010). Similarly, we show here that the C terminus in p110β is critical for lipid kinase activity.

Interestingly, the primary interaction site of p110β with the cSH2 is part of a regulatory “square” composed of helices Kα10, Kα11, and Kα12, which were described as three sides of an imaginary rectangle (Lempiäinen and Halazonetis, 2009). While the cSH2 contacts Kα11 and Kα12, the nSH2 interacts with Kα10. As this square envelops the key loops for catalysis, any changes in the conformation of the square could possibly lead to allosteric regulation of the active site, either changing the affinity for lipid substrate or facilitating phosphoryl transfer. In fact, somatic mutations in p110α in human tumors cluster on the perimeter of the square, suggesting the importance of this square in the regulation of the enzyme (Figure S5). Other proteins may also regulate PI3Ks through interactions with the regulatory square (Figure S6).

The inhibition of p110β by the cSH2 can be released upon binding to RTK phosphopeptides. However, the way phosphopeptide breaks the contact between p110 and the SH2 domains is distinct for the nSH2 and cSH2 (Movie S2). The pY-binding site on the nSH2 is buried in the interface with the catalytic subunit, thus pY binding breaks the contact by direct competition. In contrast, the pY-binding site on the cSH2 is exposed, and displacement of the cSH2 from the catalytic subunit requires five or more residues C-terminal to the pY. This means that various tyrosine-phosphorylated receptor kinases and adaptor proteins upstream of PI3Ks could have different potencies to displace the cSH2 inhibitory contact.

We find that the activity of p110β/p85-niSH2 and p110β/p85-icSH2 complexes with soluble lipid substrate as well as with water (ATPase activity in the absence of a lipid substrate) is enhanced in the presence of pY_2_. This suggests that neither the nSH2 nor the cSH2 exerts its inhibition exclusively by affecting membrane binding. At least part of the activation is due to conformational changes in the catalytic elements caused by the dislodging of the SH2 domains from the catalytic subunit upon binding to phosphopeptide. Nevertheless, the highest level of activation is observed with membrane substrates, suggesting changes in membrane binding could also contribute to maximal activation.

Overexpression of wild-type p110β causes oncogenic transformation of cells, whereas wild-type p110α does not (Kang et al., 2006). There is more than one structural feature of the catalytic subunit that could contribute to intrinsic oncogenic potential. One of them is the presence of Leu1043 in the C terminus of p110β (and p110δ), equivalent to His1047 in p110α. We have shown that the L1043H-p110β mutation decreases enzyme activity in the presence of RTK phosphopeptide. Conversely, naturally occurring mutations of this residue to a leucine in p110α display increased activity and are highly oncogenic. It seems that p110α has an intrinsic inhibitory mechanism involving His1047 at the elbow region, whereas p110β and p110δ need an extrinsic brake, provided by the cSH2, to inhibit their activity. Other structural features, such as the more basic character of the putative membrane-binding CBR loops in the C2 domain of p110β and p110δ versus p110α, could also affect oncogenic potentials. Indeed, mutations of basic residues in CBR3 of p110δ (Denley et al., 2008) or mutation of a basic residue in CBR1 of p110β (Dbouk et al., 2010) dramatically reduced their transformation potency. The iSH2 inhibitory interaction with the C2 domain can also provide a differential brake on p110 activity. It was recently shown that the iSH2-C2-mediated inhibition is more pronounced in p110α than in p110β (Dbouk et al., 2010). This constitutes an SH2-independent brake on p110 activity.

In addition to its lipid kinase activity, PI3Ks also exhibit kinase activity toward peptides (Bondeva et al., 1998) and water. The ability to bind different substrates suggests plasticity in the active site, which is reflected by the weak density of the activation loop (i.e., substrate-binding loop) in our structure. Thus, we expect that the conformation of the activation loop observed in our structure would be changed upon substrate binding.

Besides regulation by interaction with RTKs, the activity of the p110 catalytic subunit could also be modulated by the posttranslational modifications of the regulatory subunits. When we map the reported p85 modification sites on the structure, it is clear that many of the modified residues are at the interface with the catalytic subunit and could thereby influence the p110 activity (Figure S7). There are several phosphorylation sites in the cSH2. One of them is the Tyr688 in p85α, whose phosphorylation by the Src family kinases leads to upregulation of PI3K activity, and this activation is reversed by dephosphorylation of Tyr688 by Shp1 (Cuevas et al., 2001; Chan et al., 2002). Our structure shows that this tyrosine is close to the cSH2/catalytic subunit interface and suggests that its phosphorylation could disinhibit p110β.

Our work illuminates unexpected aspects of p110β inhibition by the p85 regulatory subunit and activation by RTKs. The p85 SH2 domains can contribute to the well-known isoform-dependent specificities in PI3K signaling, despite being shared by all class IA catalytic subunits. Further regulatory complexity that needs to be unraveled is the mechanism of unique regulation of p110β by Gβγ heterodimers.

## Experimental Procedures

### Constructs, Design and Cloning

Plasmids encoding p110β, p85α, p85β, and p55γ constructs shown in Table S2 were generated using the In-Fusion PCR method (Clontech-Takara Bio Europe, Saint-Germain-en-Laye, France). The p110β constructs cloned into pFastBac-HTb (Invitrogen Ltd, Paisley, UK) have an N-terminal extension encoded by the vector (MSYHHHHHHDYDIPTTENLYFQGAMDL), comprising a His_6_ tag and a TEV-protease cleavage site. The p85 and p55 constructs cloned into pFastBac1 (Invitrogen) do not have tags. Point mutations in p110β, p85, and p55 constructs shown in Table S2 were generated using the Quick-Change protocol (Agilent Technologies UK Ltd., Stockport, Cheshire, UK) and verified by sequencing.

For mammalian expression, pMIG- and pMIR-derived vectors were used (Kulathu et al., 2008). A myc-tag was inserted at the N terminus of human p110β and a FLAG-tag was inserted at the N terminus of human p85α, using standard PCR and cloning strategy (sequences MEQKLISEEDLGGSTR and MGDYKDDDDKGGSTR ahead of genes). Mutagenesis in p85α was performed in pFastBac1 vectors and subcloned into mammalian expression vectors.

### Protein Expression and Purification

A detailed procedure of protein purification is described in the Supplemental Experimental Procedures. Briefly, proteins were expressed in Sf9 cells, using recombinant baculoviruses. Cells were coinfected for 63 hr with viruses encoding the catalytic and regulatory subunits. Cells were lysed by sonication and the protein complexes were purified by sequential chromatography on HisTrap, Q-Sepharose, heparin, and gel-filtration columns.

### Crystallization

Mouse His_6_-p110β(1-1064)/p85β-icSH2(423-722) complex was diluted to 4 mg/ml, mixed with 20 mM (final concentration) sodium phenyl phosphate (Sigma P-7751) and 150 μM of the PI3K inhibitor GDC0941 (Folkes et al., 2008). The initial crystallization conditions were obtained from a broad screen of 1056 conditions (Stock et al., 2005) in 96-well MRC crystallization plates (SWISSCI AG, Zug, Switzerland). Additives (GDC0941 and phenyl phosphate) were identified by differential scanning fluorimetry (see Supplemental Experimental Procedures). Optimal crystals were obtained at 22°C in hanging drops over reservoirs of 24-well plates (Hampton Research, Aliso Viejo, CA) containing 12% polyethylene glycol 3350, 0.1 M potassium citrate at pH 6, and 0.4 M lithium sulfate. The drops contained 1 μl each of protein and reservoir solutions. The crystals were cryoprotected by stepwise addition of cryoprotectants consisting of the reservoir solution with 20 mM sodium phenyl phosphate, 150 μM of GDC0941, and an increasing concentration of glycerol up to 20% (in 5% increments). Crystals were flash frozen in liquid nitrogen.

### Data Collection and Structure Determination

See Supplemental Experimental Procedures.

### Kinase Assays

See Supplemental Experimental Procedures.

### Mammalian Cell Culture and Western Blots

See Supplemental Experimental Procedures.

## Figures and Tables

**Table 1:** Data Collection and Refinement Statistics

Data Collection	
Space group	P6_5_22
Cell dimensions	
*a*, *b*, *c* (Å)	134.3, 134.3, 428.1
α, β, γ (°)	90, 90, 120
Resolution (Å)	3.3 (3.5–3.3)
R_sym_ or R_merge_	0.099 (0.674)
*I* / σ*I*	10.4 (1.9)
Completeness (%)	98.9 (99.9)
Redundancy	5.2 (5.4)

**Refinement**	

Resolution (Å)	44–3.3 (3.4–3.3)
No. reflections	34250 (2651)
R_work_/R_free_	0.24/0.3 (0.26/0.3)
No. atoms	9848
Protein	9813
Ligand/ion	35
Water	0
B factors	129
Protein	129
Ligand/ion	97
Water	0
Rmsds	
Bond lengths (Å)	0.01
Bond angles (°)	1.33

The numbers in parentheses refer to statistics for the highest-resolution bin.

## Accession Numbers

Coordinates and structure factor amplitudes for the p110β/p85β crystal structure have been deposited in the Protein Data Bank under ID code 2y3a (Table 1).

## Supplemental Information

Document S1. Supplemental Experimental Procedures, Supplemental References, Seven Figures, and Two Tables

Movie S1. Model of PI3K Beta Activation on Membranes

Movie S2. Differential Mode of p110β Disinhibition by Phosphopeptide for the nSH2 and cSH2 Movie S2 shows a model of nSH2 and cSH2 release from p110β by pY phosphopeptides (pY shown as yellow spheres). The pY binding site on the nSH2 is at the interface with p110β, whereas the pY binding site on the cSH2 is exposed. Phosphopeptides having at least five residues following the pY are necessary to break the contact of the cSH2 with p110β.
