# Supplementary material for: Structure of Lipid Kinase p110β/p85β Elucidates an Unusual SH2-Domain-Mediated Inhibitory Mechanism
Source: Mol Cell. 2011 Mar 4;41(5):567–78. doi: 10.1016/j.molcel.2011.01.026 (PMC3670040; doi:10.1016/j.molcel.2011.01.026)
Supplement: Document S1. Supplemental Experimental Procedures, Supplemental References, Seven Figures, and Two Tables [file mmc1.pdf]

**Supplemental Information**

Molecular Cell, *Volume 41*

# Structure of Lipid Kinase p110β/p85β Elucidates Unusual SH2-Domain-Mediated Inhibitory Mechanism

Xuxiao Zhang, Oscar Vadas, Olga Perisic, Karen E. Anderson, Jonathan Clark, Phillip T. Hawkins, Len R. Stephens, and Roger L. Williams

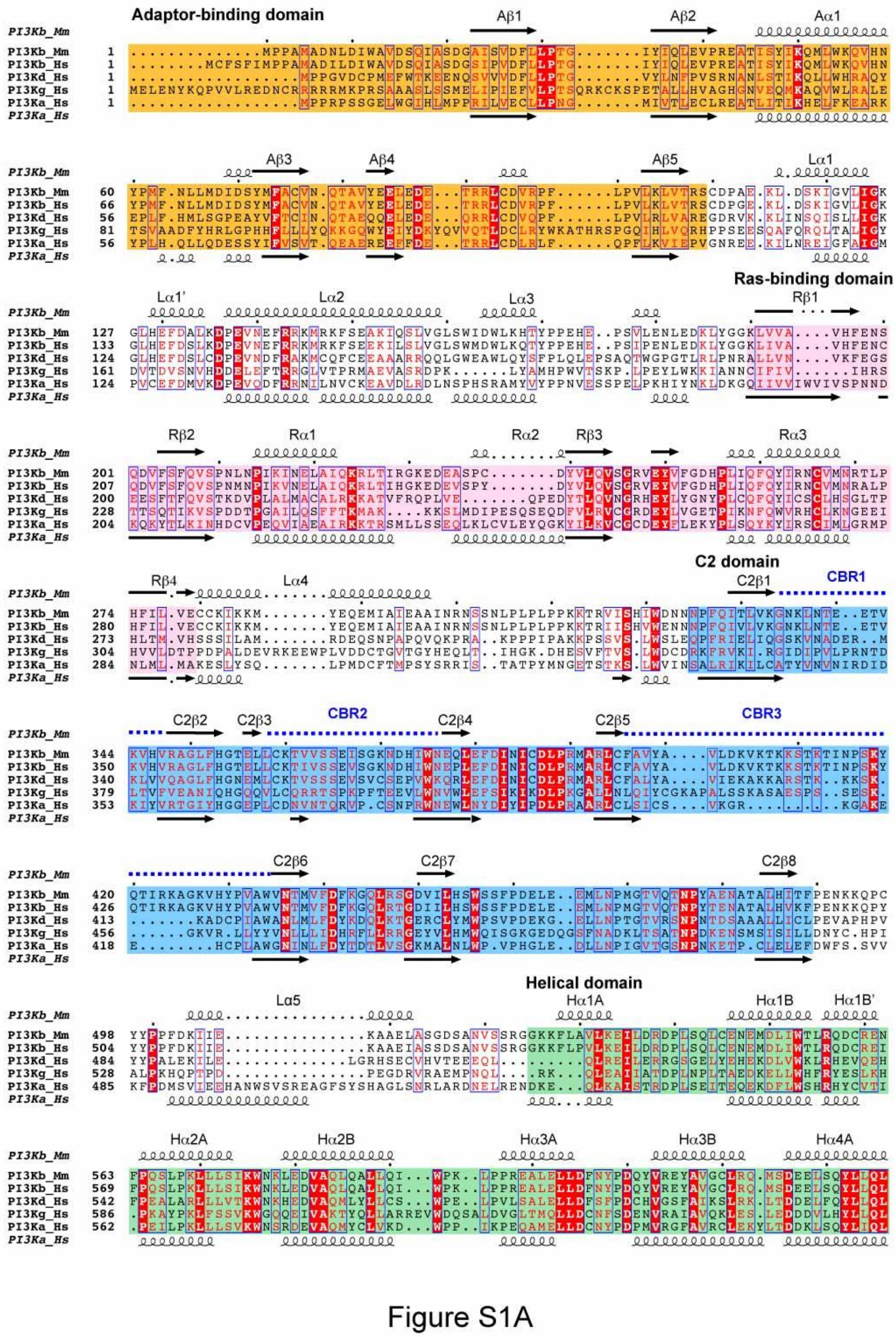
Adaptor-binding domain
Ras-binding domain
C2 domain
CBR1
CBR2
CBR3
Helical domain

Figure S1A

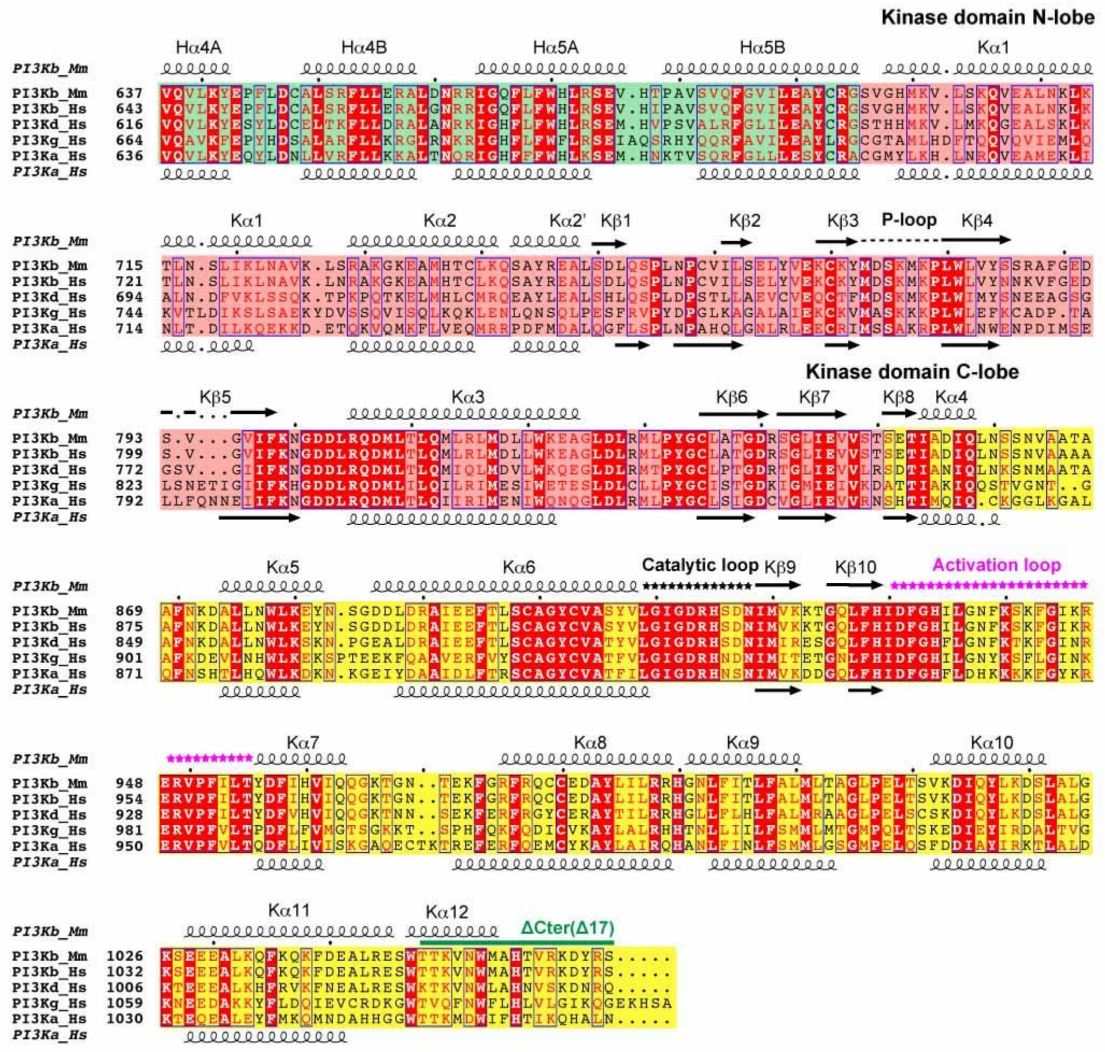


Figure S1A (continued)

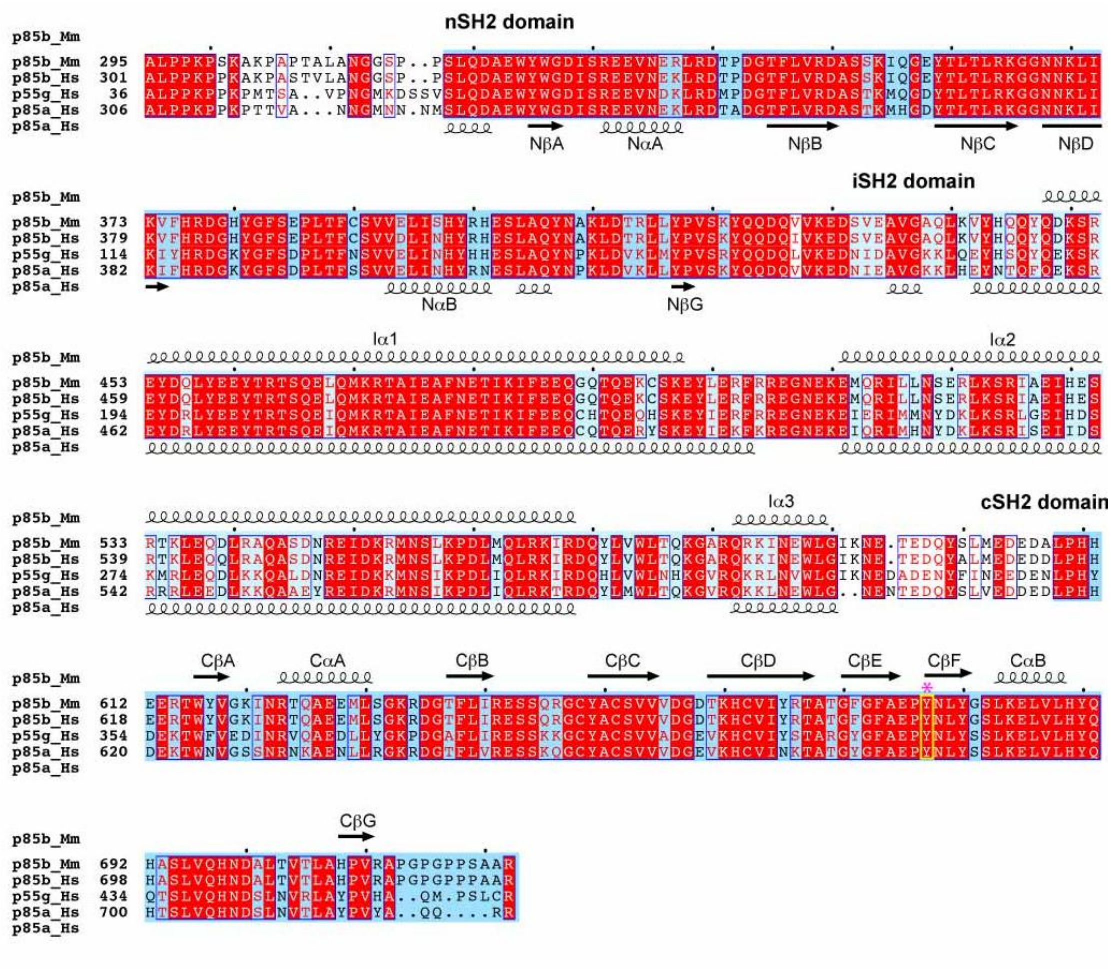


Figure S1B

**Figure S1, Related to Figure 1A. Structural Alignments of Class I p110 Catalytic and p85 Regulatory Subunits**

(A) Sequences of mouse p110β (UniProt Q8BTI9, top), human p110β (UniProt P42338), human p110δ (UniProt O00329), human p110γ (UniProt P48736) and human p110α (UniProt P42336, bottom) were aligned using ClustalW (Thompson et al., 1994). The secondary structure elements of p110β and p110α are depicted above and below the alignment, respectively. Absolutely conserved amino acids are depicted as white letters with red background. Partially conserved residues are shown in red with blue boxes. The CBRs, P-loop, the catalytic loop and the activation loop are highlighted and labeled.

(B) Sequence alignment of murine p85β (UniProt O08908), human p85β (UniProt O00459), human p85α (UniProt P27986) and human p55γ (UniProt Q92569). The secondary structure elements of p85β are labeled on top of the alignment, whereas the elements of p85α are labeled underneath. The Tyr in the cSH2 that contacts the kinase domain of p110β is marked by a star (*).

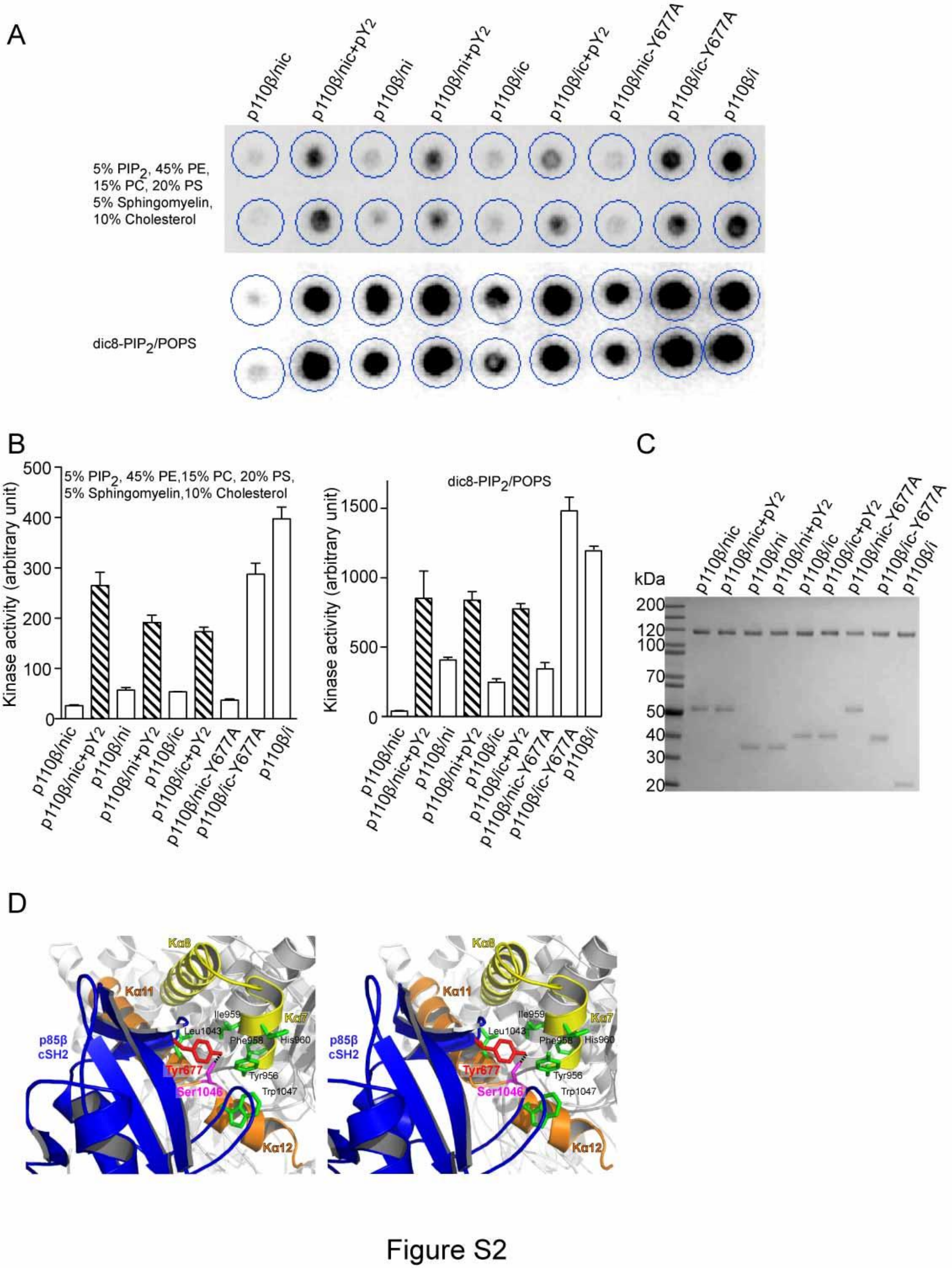


**Figure S2, Related to Figures 1 and 2. Lipid Kinase Activity of p110β in Complexes with p85β Constructs as Measured by $^{32}$P-Phosphorylation of $PIP_2$**

(A) Scan of the phosphor screen. The upper two rows are duplicates for the "brain lipids" vesicles. The lower two rows are duplicates for the diC8-$PIP_2$/POPS vesicles (Invitrogen). The reactions contained various PI3K constructs (at 5nM final concentration) in the presence

or absence of 10 μM PDGF bis-phosphopeptide. Reactions were carried out with 1 μCi [$\gamma^{32}$P]-ATP per reaction, with 100 μM ATP and 1 mg/ml total "brain lipids" or 1.5 mM diC8-$PIP_2$/POPS vesicles for 60 min. The vesicle compositions are indicated.

(B) Integrated intensities of the spots using the "brain liposomes" (left panel) or diC8-$PIP_2$/POPS liposomes (Invitrogen) (right panel), quantitated with the ImageQuant software. The bars represent standard deviations of the mean for two measurements. The hatched bars are for reactions in the presence of PDGF bis-phosphopeptide.

(C) SDS PAGE analysis of the proteins used for the assays in (A and B). Samples of 10 μl of 1.7 nM protein solutions for each of the reaction were analyzed on a 4-12% NuPAGE Novex Bis-Tris gel in MES running buffer (Invitrogen). The upper band is the p110 subunit and the lower band is the regulatory subunit.

(D) Stereo view of the contacts between the p85β-cSH2 domain and the C-lobe of the kinase domain. The Tyr677-p85β (red) inserts into a hydrophobic groove between the two elbows kα7/kα8 (yellow) and kα11/kα12 (orange). Tyr677-p85β also makes a potential hydrogen bond with Ser1046-p110β (dashed black line).

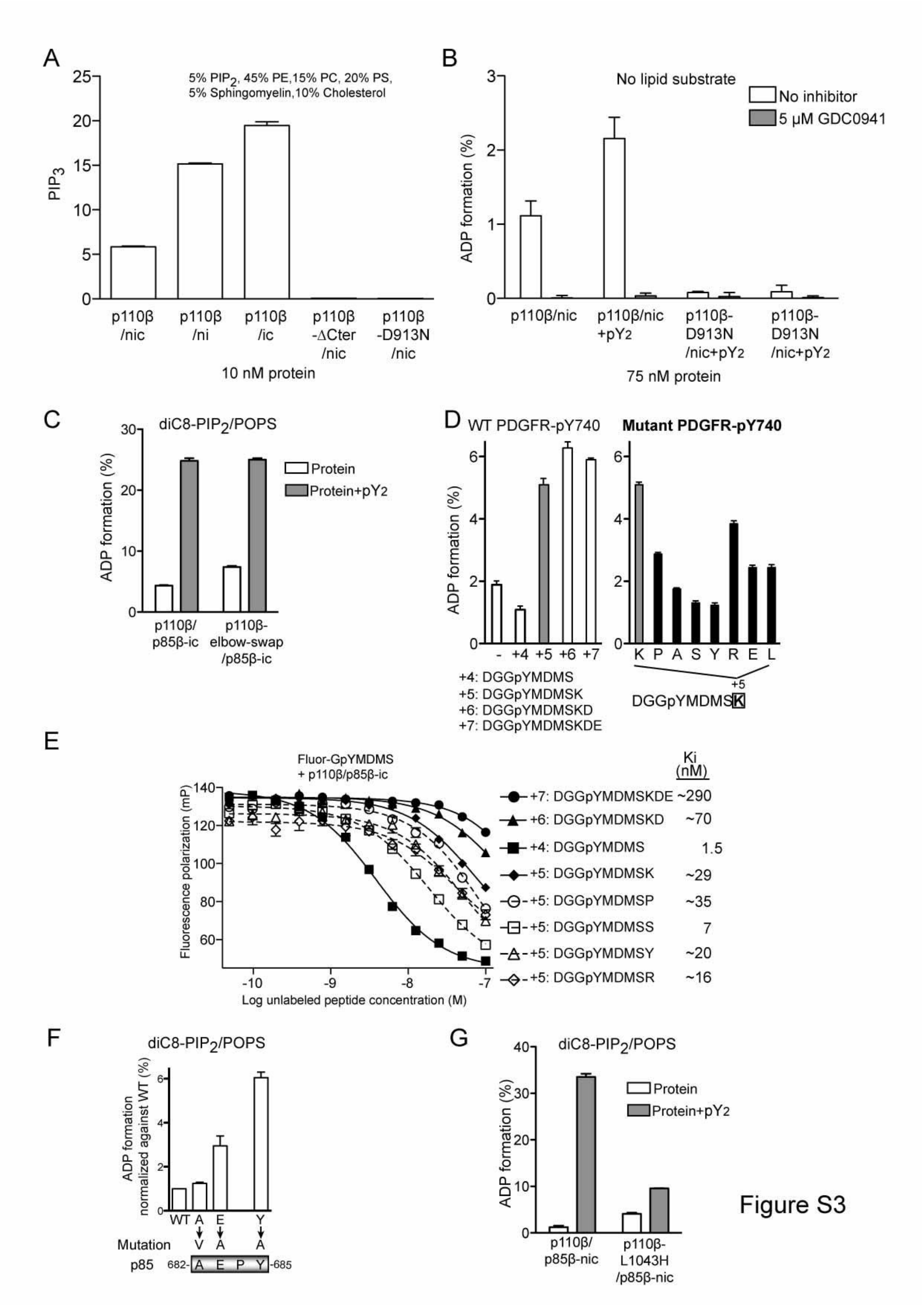


**Figure S3, Related to Figures 1 and 2. Lipid Kinase Assays for p110β/p85 Constructs**

(A) Lipid kinase activity of the p110β/p85β-nic, p110β/p85β-ni and p110β/p85β-ic, p110β C-terminal truncation/p85β-nic (ΔCter) and kinase dead (D913N) constructs as determined by mass spectroscopy. Error bars in panel A and other panels indicate SEM of four replicates.

Vesicles were "brain lipids" as in Figure S2. The protein concentration was 10 nM with 100 μM ATP and 1 mg/ml lipid. Reactions were carried out for 60 min. The bars show the integrated areas for the stearoyl-arachidonyl PtdIns(3,4,5)$P_3$ peak.

(B) ATPase activity (no lipid substrate) of the wild-type and kinase dead p110β/p85β-nic construct in the absence and presence of 10 μM PDGF bis-phosphopeptide. Protein concentration was 75 nM with 100 μM ATP. Shaded bars represent activity for reactions with 5 μM added PI3K inhibitor GDC0941.

(C) Lipid kinase activity of a p110β/p85-icSH2 complex in which p110β Kα7/Kα8 elbow (966-KTGN) was replaced by the elbow of p110α (968-AQECTK).

(D) Effect of different RTK phosphopeptides (10 μM) on activity of p110β/p85β-icSH2 complexes (1 nM), showing release of the cSH2 inhibition by the peptide is both length and sequence dependent. Left panel: wild-type pY+4, pY+5, pY+6 and pY+7 phosphopeptides from human PDGFR showed that peptides longer than pY+4 are required for disinhibition of the p110β/p85β-icSH2 complex. Right panel: the PDGF phosphopeptide mutations at the pY+5 position affect ability of the peptide to disinhibit enzyme activity.

(E) Binding of different PDGFR phosphopeptides to p110β/p85β-icSH2 complex (6 nM) was tested by competition with fluorescein-labeled PDGFR phosphopeptide (Fluor-GpYMDMS, 2 nM). The Kd of the Fluor-peptide for the p110β/p85β-icSH2 complex is 1.3 nM (titration not shown).

(F) Effect of p85 mutations on enzyme activity (Transcreener assay of ADP formation). Effect of mutations in the Ala-Glu-Pro-Tyr loop of the p85-cSH2 on p110β activity (normalized to the wild-type complex).

(G) Lipid kinase activity of the p110β-L1043H mutant in a complex with p85β-nicSH2 compared with the wild-type complex in the absence and presence of 10 μM PDGFR bis-phosphopeptide (pY2).

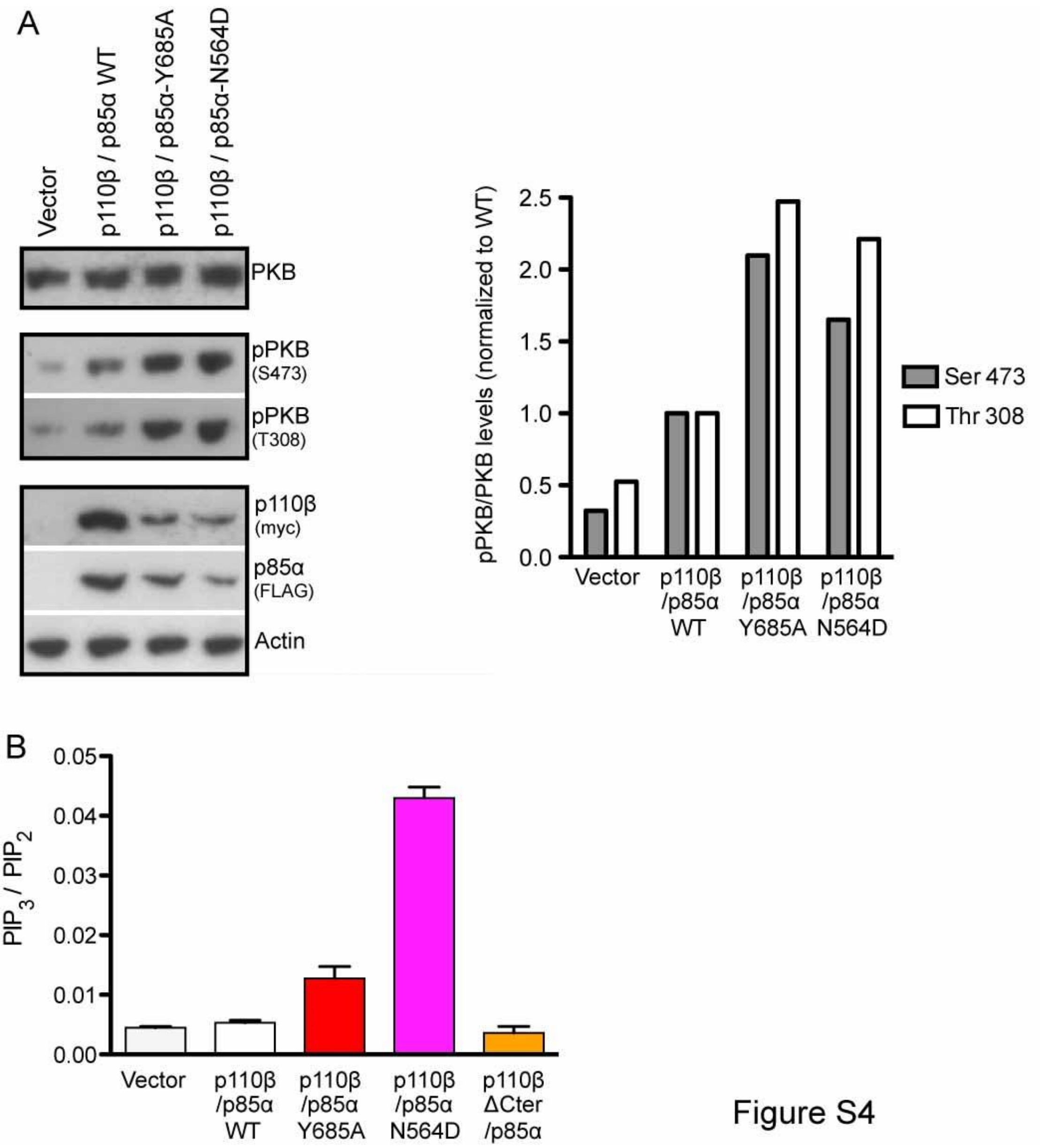


**Figure S4, Related to Figure 4. Tyr-Mutation in cSH2 Activates PI3K Downstream Signaling in Cells**

(A) Western blot of HEK cells over-expressing wild-type and mutant human p110β/p85α complexes. Protein kinase B (PKB) phosphorylation (pPKB) levels at residues Ser473 and Thr308 were quantified and normalized WT p110β/p85α (WT).

(B) Quantification of PtdIns(3,4,5)$P_3$ levels in HEK cells over-expressing wild-type and mutant human p110β/p85α complexes. Phospholipids were extracted from cells and PtdIns(3,4,5)$P_3$ and PtdIns(4,5)$P_2$ levels were quantified by mass spectrometry. Graph shows ratio of PtdIns(3,4,5)$P_3$ / PtdIns(3,4,5)$P_2$ for each construct for two separate experiments measured in duplicates. Error bars indicate SEM.

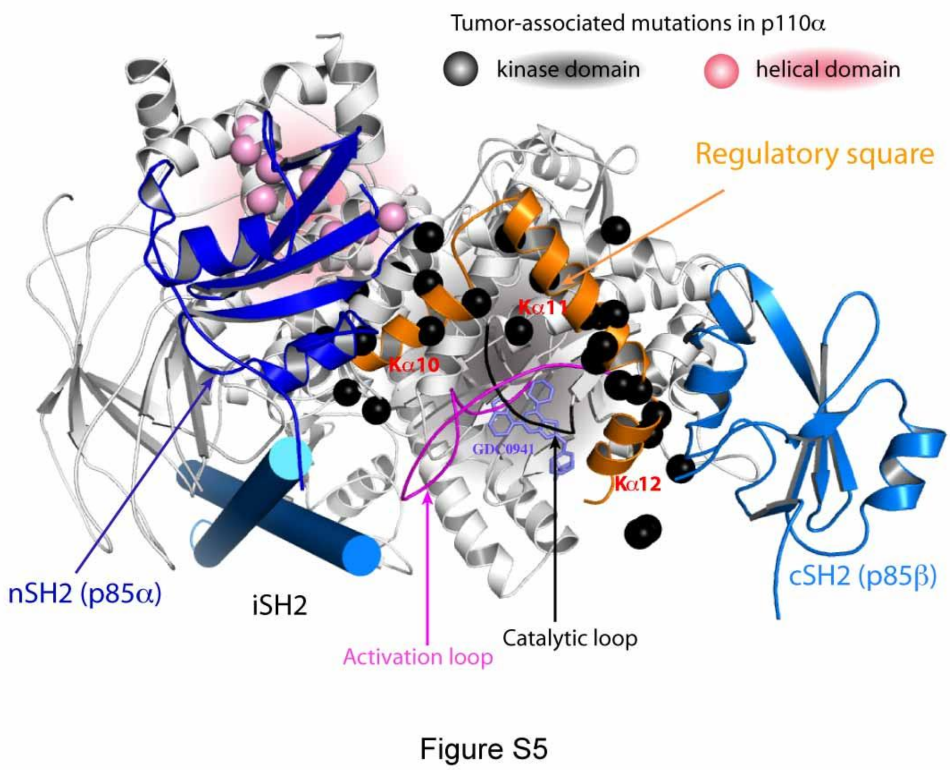


**Figure S5. SH2 Domain Mediated Inhibition and Oncogenic Activation May Operate through the Same Structural Elements**

A 'regulatory square' formed by helices Kα10, Kα11 and Kα12 (orange) surrounds the critical catalytic elements, catalytic loop (black) and activation loop (magenta). To illustrate the location of somatic mutations in the p110α kinase and helical domains, the p110α/p85α-niSH2 structure (PDB ID: 3HHM) was superimposed on p110β/p85β-icSH2 and black and pink spheres represent the Cα positions of the p110α mutations.

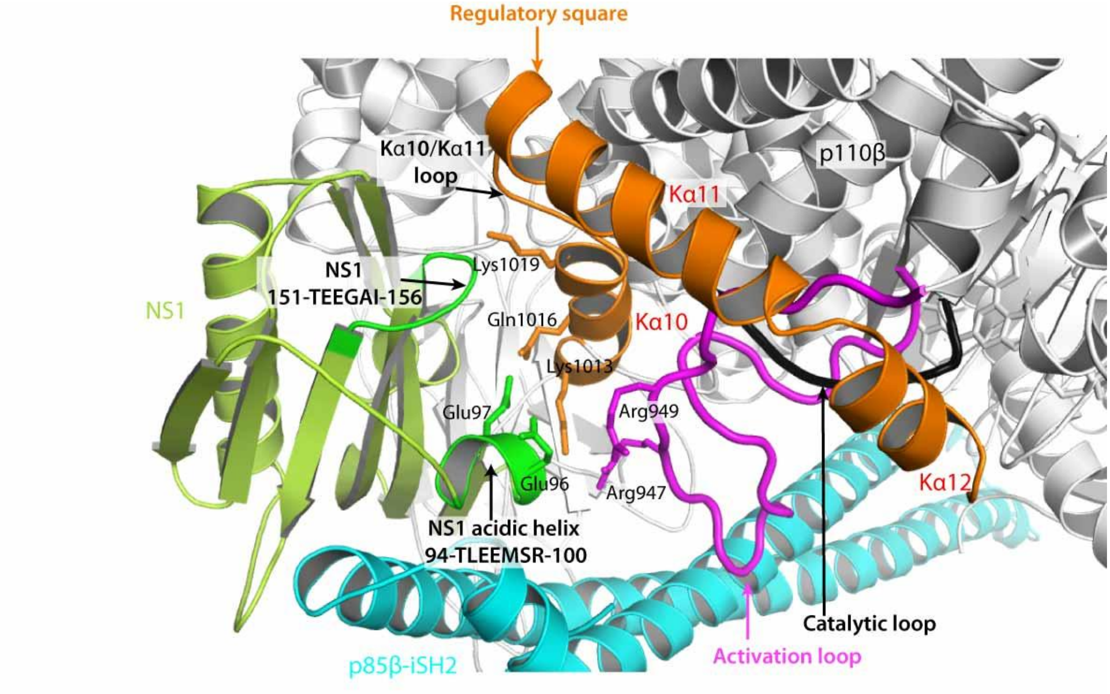


Figure S6

**Figure S6. NS1-Mediated Activation of PI3K**

Superposition of the influenza A nonstructural protein (NS1) effector domain (ED)/p85β-iSH2 (PDB ID: 3L4Q) on the p110α/p85α structure showed that the NS1 would displace the inhibitory contact with the nSH2 (Hale et al., 2010). Superposition of the NS1 complex on our p110β/p85β suggests a probable contact with the regulatory square. The acidic helix of NS1 (94-TLEEMSR-100), which is important for PI3K activation during viral infection, may interact with the activation loop (Arg947 and Arg949) and Kα10. In addition, a loop in NS1 (151-TEEGAI-156) would be in a close vicinity to the Kα10 and Kα10/Kα11 loop (1024-LGK-1026). Therefore, NS1 may activate p110 by sterically preventing inhibition by the nSH2 and possibly through direct, activating interactions with elements of the regulatory square.

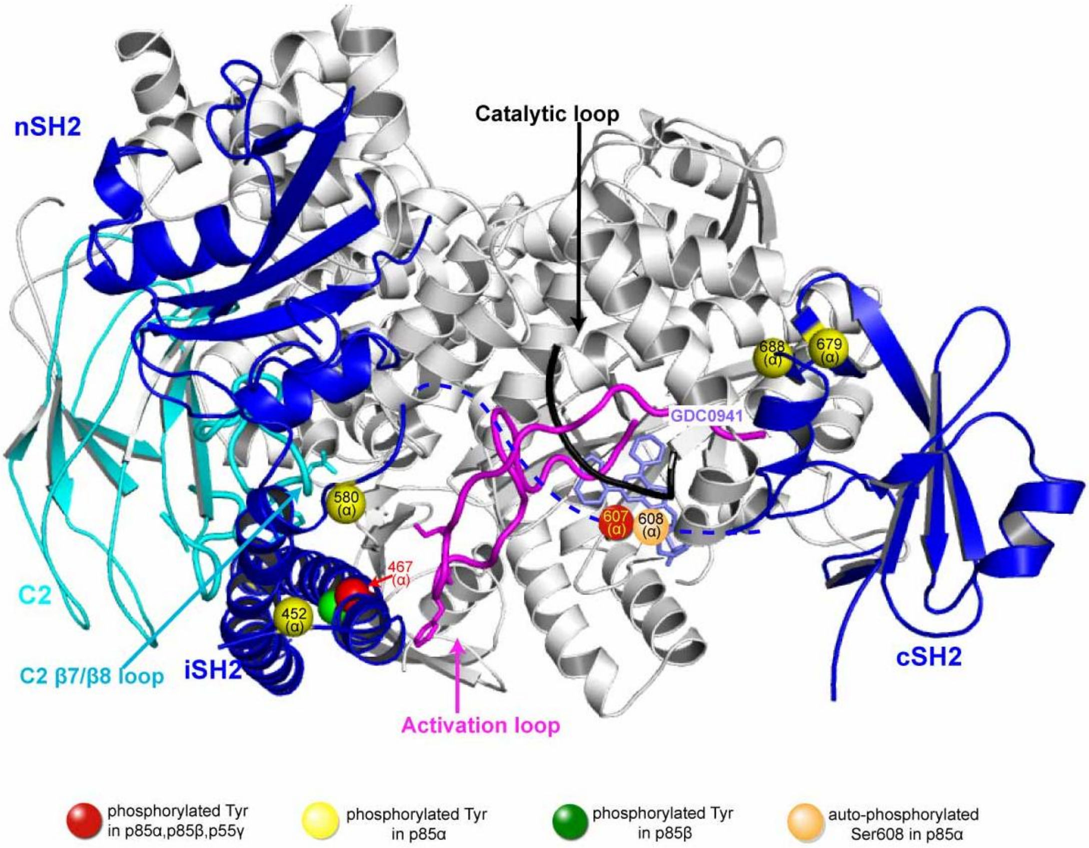


| Domain | Residues | Hsp85α | | Hsp85β | | Mmp85β | | Hsp55γ |
|---|---|---|---|---|---|---|---|---|
| | | *experimetal* | *by similarity* | *experimetal* | *by similarity* | *experimetal* | *by similarity* | *experimetal* |
| BH | Thr | 152 | | | / | | / | |
| | Ser | 154 | | | / | | / | |
| | Ser | | / | 262 | | | / | |
| | Ser | | / | 263 | | | / | |
| nSH2 | Tyr | | 368 | 365 | | | 359 | |
| iSH2 | Tyr | 452 | | | 449 | | 443 | |
| | Tyr | 467 | | 464 | | 458 | | 199 |
| | Tyr | 508 | | | 505 | | 499 | |
| | N6-acetyllysine | 530 | | | / | | / | |
| | Tyr | | 556 | 467 | | 461 | | |
| | Tyr | 580 | | | 577 | | 571 | |
| icSH2linker | Tyr | 607 | | 605 | | 599 | | |
| cSH2 | Tyr | 679 | | | 677(Phe) | | 671(Phe) | |
| | Tyr (by Src) | 688 | | | 686 | | 680 | |

Figure S7

**Figure S7. Posttranslational Modifications of the Regulatory Subunit**

The residues undergoing phosphorylation are highlighted as colored spheres (http://www.uniprot.org/uniprot/). The phosphorylated residues from all three isoforms are mapped on the structure of Mmp110β/p85β-icSH2 complex. The residue numbering for yellow and red spheres refers to p85α. The dashed line (blue) is the disordered iSH2/cSH2 linker.

The phosphorylated residues could affect the activity of the complex. For example, a conserved tyrosine in Iα1 of the iSH2 undergoes phosphorylation in all three regulatory

subunits (Tyr467-Hsp85α, Tyr464-Hsp85β, Tyr199-Hsp55γ, Tyr458-Mmp85β) (as listed in UniProt entries P27986, O08908, O00459, Q92569). This tyrosine is in close proximity to both the activation loop and the C2 β7/β8 loop and its phosphorylation could potentially affect the kinase activity. Another conserved Tyr modified by phosphorylation is in the iSH2-cSH2 linker (Tyr607-Hsp85α, Tyr605-Hsp85β, Tyr341-Hsp55γ, Tyr599-Mmp85β). Interestingly, this Tyr is just before Ser608 in Hsp85α, which is known to be auto-phosphorylated by the p110 catalytic subunit, resulting in lower enzymatic activity (Foukas et al., 2004). Tyr phosphorylation may have a similar effect as Ser608 or it may influence the status of Ser608 phosphorylation.

Besides the phosphorylation sites shared by all regulatory subunits, there are several phosphorylated sites unique to p85β relative to p85α, one being Tyr467-Hsp85β in Iα1 of iSH2 that contacts the C2 β7/β8 loop. The modification of this tyrosine could potentially influence the interaction between p85-iSH2 and the catalytic domain. It will be interesting to see whether the p85 phosphorylation sites are targeted by PTEN (phosphatase and tensin homolog), a tumor suppressor commonly mutated in cancers that was shown to associate with p85 during kinase activation (Barber et al., 2006; Rabinovsky et al., 2009) and proposed to dephosphorylate p85β (He et al., 2010).

**Table S1. Comparison of GDC0941 Contacts with p110 Subunits**

The contacts between the bound GDC0941 and catalytic subunits are summarized for the p110β, p110δ and p110γ isotypes. An ‘x’ marks the presence of a contact (distance less than 4 Å). Potential hydrogen bonds are also indicated.

| p110 isoform | | | Compounds/p110 isoform | | |
|---|---|---|---|---|---|
| **Mmp110β** | **Hsp110α** | **Hsp110γ** | **GDC0941 (p110β)** PDB ID: 2Y3A | **GDC0941 (p110δ)** PDB ID: 2WXP | **GDC0941 (p110γ)** PDB ID: 3DBS |
| Arg729 | Lys708 | Ser760 | | x | |
| Lys771 | Thr750 | Lys802 | | | x H-bond O1 |
| Tyr772 | Phe751 | Val803 | x | x | |
| Met773 | Met752 | Met804 | x | x | x |
| Asp774 | Asp753 | Ala805 | x | x H-bond O2 | x H-bond O2 |
| Trp781 | Trp760 | Trp812 | | x | |
| Ile797 | Ile777 | Ile831 | | x | x |
| Lys799 | Lys779 | Lys833 | x | x | x |
| Asp802 | Asp782 | Asp836 | | | x |
| Asp807 | Asp787 | Asp841 | x H-bond N6 | x H-bond N6 | x H-bond N6 |
| Tyr833 | Tyr813 | Tyr867 | x H-bond N7 | x | x H-bond N7 |
| Ile845 | Ile825 | Ile879 | x | x | x |
| Glu846 | Glu826 | Glu880 | x | x | x |
| Val847 | Val827 | Ile881 | x | x | x |
| Val848 | Val828 | Val882 | x H-bond O3 | x H-bond O3 | x H-bond O3 |
| Ser851 | Ser831 | Ala885 | | x | |
| Thr853 | Thr833 | Thr887 | | x | x |
| Met920 | Met900 | Met953 | x | x | x |
| Phe928 | Phe908 | Phe961 | | x | |
| Ile930 | Ile910 | Ile963 | x | x | x |
| Asp931 | Asp911 | Asp964 | x | x | |

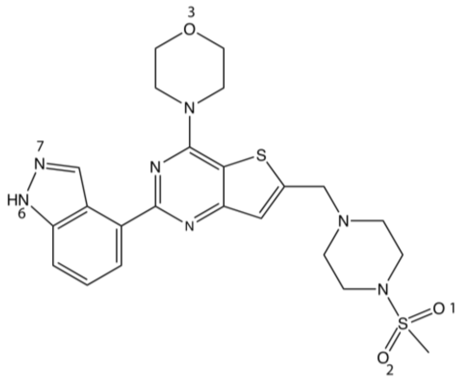

## Table S2. Cloning and Constructs

| Protein Accession No | Construct (residues) | Forward Primer | Reverse Primer | Template | Vector | Restriction sites flanking the insert | Internal ID |
|---|---|---|---|---|---|---|---|
| Q8BTI9 | Mmp110β-full length (1-1064) | CAGGGCGCCATGGGATCCATGCCTCCTGCTATGGCAGAC | GGTACCGCATGCCTCGAGCTAGGACCTGTAGTCTTTCCG | RZPD: IRAKp961H1423Q2 | pFastBac-HTb | BamHI/XhoI | pOP561 |
| | Mmp110β-ΔCter (1-1047; ΔC17) | GTATTTTCAGGGCGCCATGGGATCCATGCCTCCTGCTATGGCAGACAACC | GCTTGGTACCGCATGCCTCGAGCTACCAGCTTTCCCTGAGGGCCTCG | Mmp110β-(1-1064) | pFastBac-HTb | BamHI/XhoI | pOV2 |
| | Mmp110β-full length (1-1064)-D913N | GTCCTCGGCATTGGTAACAGGCACAGTGACAAC | GTTGTCACTGTGCCTGTTACCAATGCCGAGGAC | Mmp110β-(1-1064) | pFastBac-HTb | BamHI/XhoI | pXZ73 |
| | Mmp110β-full length (1-1064)-L1043H | GCAGAAGTTTGACGAGGCCCACAGGGAAAGCTGGACTAC | GTAGTCCAGCTTTCCCTGTGGGCCTCGTCAAACTTCTGC | Mmp110β-(1-1064) | pFastBac-HTb | BamHI/XhoI | pOV3 |
| | Mmp110β-(1-109)-tev-(110-1064) | CTCAAACTAGTGACTAGAAGCGAAAACCTGTATTTTCAGGGCGGGGACCCCGCAGAAAAATTGGAC | GTCCAATTTTTCTGCGGGGTCCCCGCCCTGAAAATACAGGTTTTCGCTTCTAGTCACTAGTTTGAG | RZPD: IRAKp961H1423Q2 | pFastBac-HTb | BamHI/XhoI | pOP562 |
| P42338 | Hsp110β-full length (1-1070) | GTATTTTCAGGGCGCCATGGGATCCATGTGCTTCAGTTTCATAATGCCTCC | GGTACCGCATGCCTCGAGCTAGGACCTGTAGTCTTTCCGAAC | IMAGE: 40008544 | pFastBac-HTb | BamHI/XhoI | pXZ107 |
| | Hsp110β-full length (1-1070) | ATGGAGCAGAAACTCATCTCTGAAGAGGATCTGGGCGGATCCACGCGTATGTGCTTCAGTTTCATAATGCCTCCTGCTATGGCAGAC + CTAGGCGCCGGAATTAGATCTCTCGAGGCCACCATGGAGCAGAAACTCATCTCTGAAGAGGATCTGGGCG | TAGGGGGGGGGGGCGGAATTCGCGGCCGCCTAGGACCTGTAGTCTTTCCGAACTGTGTGGGC | Hsp110β-(1-1070) | pMIG | XhoI/EcoR1 | pOV34 |
| | Hsp110β-ΔCter (1-1053; ΔC17) | ATGGAGCAGAAACTCATCTCTGAAGAGGATCTGGGCGGATCCACGCGTATGTGCTTCAGTTTCATAATGCCTCCTGCTATGGCAGAC | TAGGGGGGGGGGGCGGAATTCGCGGCCGCCTACCAGCTTTCCCTGAGCGCCTCATC | Hsp110β-(1-1070) | pMIG | MluI/EcoRI | pOV35 |
| O08908 | Mmp85β-nicSH2(318-722) | ACCGTCCCACCATCGGGCGCGGATCCATGTCGCTTCAGGATGCAGAGTGGTACTG | AGGCTCTAGATTCGAAAGCGGCCGCGCTAGCGTGCTGCAGACGGTGGGCC | IMAGE: 2581969 | pFastBac1 | BamHI/NotI | pXZ83 |
| | Mmp85β-niSH2(318-599) | ACCGTCCCACCATCGGGCGCGGATCCATGTCGCTTCAGGATGCAGAGTGGTACTG | AGGCTCTAGATTCGAAAGCGGCCGCGCTAATACTGGTCCTCAGTCTCGTTCTTGATTCCC | IMAGE: 2581969 | pFastBac1 | BamHI/NotI | pXZ71 |
| | Mmp85β-iSH2(423-599) | ACCATCGGGCGCGGATCCATGTCGCAACAAGACCAGGTGGTGAAGGAGGAC | CTAGATTCGAAAGCGGCCGCGCTAATACTGGTCCTCAGTCTCGTTCTTGATTCCC | IMAGE: 2581969 | pFastBac1 | BamHI/NotI | pXZ72 |
| | Mmp85β-icSH2(423-722) | ACCGTCCCACCATCGGGCGCGGATCCATGTCGCAACAAGACCAGGTGGTGAAGGAGGA | AGGCTCTAGATTCGAAAGCGGCCGCGCTAGCGTGCTGCAGACGGTGGGCC | IMAGE: 2581969 | pFastBac1 | BamHI/NotI | pXZ73 |
| | Mmp85β-icSH2(423-722)-Y677A | CGGCTTCGCAGAGCCCGCAAACCTGTACGGGTCCCTGAAGG | CCTTCAGGGACCCGTACAGGTTTGCGGGCTCTGCGAAGCCG | Mmp85β-icSH2(423-722) | pFastBac1 | BamHI/NotI | pXZ104 |
| | Mmp85β-icSH2(423-722)-E675A | CCACCGGCTTCGGCTTCGCAGCGCCCTATAACCTGTACGG | CCGTACAGGTTATAGGGCGCTGCGAAGCCGAAGCCGGTGG | Mmp85β-icSH2(423-722) | pFastBac1 | BamHI/NotI | pXZ86 |
| P27986 | Hsp85α-full length (1-724) | CCCACCATCGGGCGCGGATCCATGAGTGCTGAGGGGTACCAGTACAG | AGGCTCTAGATTCGAAAGCGGCCGCGTCATCGCCTCTGCTGTGCATATAC | IMAGE: 30528412 | pFastBac1 | BamHI/NotI | pXZ99 |
| | Hsp85α-full length (1-724) | GGCGACTACAAGGACGACGACGACAAGGGCGGATCCACG | AACCATGGTACCCGGGAATTCGCGGCCGCTCATCGCCTC | IMAGE: 30528412 | pMIR | XhoI/EcoRI | pOV29 |

| | | | | | | | |
|---|---|---|---|---|---|---|---|
| | | CGTATGAGTGCTG AGGGGTACCAGTA CAGAGCG + CTAGGCGCCGGAA TTAGATCTCTCGAG GCCACCATGGGCG ACTACAAGGACGA CGACGACAAGGG | TGCTGTGCATATAC TGGGTAGGC | | | | |
| | Hsp85α-full length (1-724)- Y685A | GGCTATGGCTTTG CCGAGCCCGCTAA CTTGTACAGCTCTC TGAAAG | CTTTCAGAGAGCT GTACAAGTTAGCG GGCTCGGCAAAGC CATAGCC | Hsp85α-full length (1-724) | pMIR | MluI/EcoR1 | pOV31 |
| | Hsp85α-full length (1-724)- N564D | CGAGAAATTGACA AACGTATGGATAG CATTAAACCAGAC CTTATCC | GGATAAGGTCTGG TTTAATGCTATCCA TACGTTTGTCAATT TCTCG | Hsp85α-full length (1-724) | pMIR | MluI/EcoR1 | pOV45 |
| | Hsp85α-icSH2(432-724) | ACCGTCCCACCAT CGGGCGCGGATCC ATGTCGCAACAGG ATCAAGTTGTCAAA GAAG | AGGCTCTAGATTC GAAAGCGGCCGCG TCATCGCCTCTGC TGTGCATATAC | IMAGE: 30528412 | pFastBac1 | BamHI/NotI | pXZ93 |
| | Hsp85α-icSH2(432-724)-Y685A | GGCTATGGCTTTG CCGAGCCCGCTAA CTTGTACAGCTCTC TGAAAG | CTTTCAGAGAGCT GTACAAGTTAGCG GGCTCGGCAAAGC CATAGCC | Hsp85α-icSH2(432-724) | pFastBac1 | BamHI/NotI | pXZ98 |
| | Hsp85α-icSH2(432-724)-A682V | CTGGCTATGGCTT TGTAGAGCCCTAT AACTTGTACAGC | GCTGTACAAGTTAT AGGGCTCTACAAA GCCATAGCCAG | Hsp85α-icSH2(432-724) | pFastBac1 | BamHI/NotI | pXZ149 |
| | Hsp85α-iSH2(431-600) | CGCGGATCCACCA TGTACCAACAGGA TCAAGTTGTCAAAG | TGCTCTAGATCAAT TGCCCAACCACTC GTTCAACTTC | IMAGE: 4290954 | pVL1393 | BamHI/XbaI | AT6 |
| O00459 | Hsp85β-icSH2(429-728) | CCCACCATCGGGC GCGGATCCATGTC GCAGCAGGACCAG ATTGTC | GGCTCTAGATTCG AAAGCGGCCGCTC AGCGGGCGGCAG GCGGCG | IMAGE: 6341766 | pFastBac1 | BamHI/NotI | pXZ106 |
| | Hsp85β-icSH2(429-728)-Y683A | CGGCTTCGCGGAG CCCGCCAACCTGT ACGGG | AGCGACCCGTACA GGTTGGCGGGCTC CGCG | Hsp85β-icSH2(429-728) | pFastBac1 | BamHI/NotI | pXZ111 |
| Q92569 | Hsp55γ-icSH2(164-461) | CCCACCATCGGGC GCGGATCCATGTC GCAACAGGATCAG TTGGTAAAAG | CAAGCTTGGTACC GCATGCCTCGAGT TATCTGCAAAGCG AGGGCATCTG | RZPD: IRATp970D05 37D6 | pFastBac1 | BamHI/XhoI | pOP646 |
| | Hsp55γ-icSH2(164-461)-Y419A | GCTATGGCTTTGC AGAGCCCGCCAAC CTGTACAGCTCTCT GAAG | CTTCAGAGAGCTG TACAGGTTGGCGG GCTCTGCAAAGCC ATAGC | Hsp55γ-icSH2(164-461) | pFastBac1 | BamHI/XhoI | pOP649 |

**Movie S1, Related to Figure 7. Model of PI3K Beta Activation on Membranes**

**Movie S2, Related to Figure 2D and 2E. Differential Mode of p110β Disinhibition by Phosphopeptide for the nSH2 and cSH2**

Model of nSH2 and cSH2 release from p110β by pY phosphopeptides (pY shown as yellow spheres) . The pY binding site on the nSH2 is at the interface with p110β, whereas the pY binding site on the cSH2 is exposed. Phosphopeptides having at least 5 residues following the pY are necessary to break the contact of the cSH2 with p110β.

**Supplemental Experimental Procedures**

**Protein Expression and Purification**

Recombinant baculoviruses were generated and propagated using the Bac-to-Bac expression system according to manufacturer's recommendations (Invitrogen). For expression, 3L of *Spodoptera frugiperda* (Sf9) cells at the density of $1.0x10^6$ cells/mL were co-infected with an optimized ratio of viruses encoding complexes of the catalytic and regulatory subunit. After 63 hr infection at 27˚C, cells were harvested and washed with ice-cold phosphate-buffered saline (PBS) supplemented with 0.5 mM AEBSF (Melford). Subsequently, cells were lysed by sonication for 4 min in 120 ml buffer A1 (20 mM Tris pH 8, 300 mM NaCl, 10 mM imidazole) containing 0.5 mM AEBSF and centrifuged for 20 min at 35,000 rpm. The supernatant was filtered through a 0.45 µm Minisart filter unit (Sartorius Biotech) before loading onto two connected 5 ml HisTrap FF columns (GE Healthcare). The columns were washed first with buffer A1, then with buffer A2 (20 mM Tris pH8, 100 mM NaCl, 10 mM imidazole, 2 mM 2-mercaptoethanol), and eluted with a gradient from 0 to 100% of buffer B (20 mM Tris pH8, 100 mM NaCl, 300 mM imidazole, 2 mM 2-mercaptoethanol). Fractions were analyzed on 4-12% Bis-Tris Novex gel (Invitrogen) using MOPS buffer. The protein complex was further purified on a HiLoad 16/10 Q Sepharose column (GE Healthcare) and on a 5 ml HiTrap Heparin HP column (GE Healthcare). Both of these columns were washed with buffer C (20 mM Tris pH 8, 100 mM NaCl, 2 mM DTT) and eluted with buffer D (20mM Tris pH 8, 2 mM DTT, 1 M NaCl). Subsequently, the complex was concentrated to 1.5 ml with AMICON 50K centrifugal filters (Millipore), loaded onto a 16/60 Superdex 200 gel filtration column (GE Healthcare) and eluted at 4˚C with buffer E (20 mM Tris pH 7.5, 100 mM NaCl, 5 mM DTT). The heterodimer was concentrated to 9 mg/ml, frozen in liquid nitrogen and stored at -80˚C.

**Cloning and Protein Purification for ΔABD-p110β**

The construct of an ABD-truncated version of p110β (ΔABD-p110β) was made using a strategy described previously for generation of ΔABD-p110δ, in which a TEV protease cleavage site is inserted in the linker region between ABD and RBD (Berndt et al., 2010). The correct insertion of the TEV site was confirmed by DNA sequencing (amino acid sequence:

105-LVTRS-(109)-ENLYFQG-(110)-GDPAK-115). For expression, the iSH2 fragment of the human p85α (residues 431-600), tagged with an N-terminal non-cleavable $His_6$ tag was used for co-infection with the catalytic subunit. For purification, cells were sonicated in lysis buffer (20 mM Tris pH8, 100 mM NaCl, 5% (v/v) glycerol, 2 mM $MgSO_4$, 10 mM imidazole and 2 mM β-mercaptoethanol). The lysate was then centrifuged, filtered and loaded onto two 5ml HisTrap FF columns (GE Healthcare). The columns were washed with lysis buffer and eluted with a gradient of 20-200 mM imidazole in lysis buffer. Fractions of p110β/p85β-iSH2 complex were pooled and loaded onto a 5-ml Heparin column equilibrated with buffer Hep-A (20 mM Tris pH 8, 100 mM NaCl, 5%(v/v) glycerol, 2 mM β-mercaptoethanol). The column was washed and eluted with a gradient of buffer Hep-B (20 mM Tris pH 8, 1 M NaCl, 5%(v/v) glycerol, 2 mM β-mercaptoethanol). This chromatography enabled the separation of excess $His_6$-tagged iSH2 (earlier peak) from the p110β/p85β-iSH2 complex (later peak). The p110β/p85β-iSH2 fractions were pooled and adjusted to 5 mM β-mercaptoethanol. TEV protease at a w/w ratio of 1:10 was added, and the mixture was incubated overnight at 4 °C. After cleavage was complete, the solution was adjusted to 1 M NaCl and loaded onto a 5 ml HisTrap column equilibrated with buffer Ni-A (20 mM Tris pH8, 1 M NaCl, 5% (v/v) glycerol, 2 mM $MgSO_4$ and 2 mM β-mercaptoethanol). The column was eluted with buffer Ni-B (20 mM Tris pH8, 1 M NaCl, 5% (v/v) glycerol, 2 mM $MgSO_4$, 2 mM β-mercaptoethanol and 200 mM imidazole). This step separates the HisTrap bound ABD/$His_6$-iSH2 from the ΔABDp110β in the flow through. The protein was then concentrated and subjected to gel filtration as described above. Protein was eluted in gel filtration buffer (20 mM Tris pH 7.5, 1% ethylene glycol, 50 mM $(NH_4)_2SO_4$, 1% (w/v) betaine, 0.02% (w/v) CHAPS and 1mM TCEP) and frozen at 5.3 mg/ml.

**Data Collection and Structure Determination**

Diffraction data were collected at the ESRF beamlines ID14-4, ID23-1 and ID29. Images were processed using IMOSFLM (Leslie, 2006) and scaled with SCALA (CCP4, 1994).

The crystal structure was solved by molecular replacement using PHASER (McCoy, 2007) with the previously published p110δ structure (PDB ID: 2WXG) and the iSH2 structure (PDB ID: 2RD0) as the search models and subsequently refined using REFMAC (Murshudov et al.,

1997). COOT (Emsley and Cowtan, 2004) was used to manually place the known cSH2 structure (PDB ID: 1QAD) in the 2mFo-DFc electron density of the map. Refinement using BUSTER (Blanc et al., 2004) was iterated with manual re-building using COOT until the structure converged.

Final statistics for the 3.3 Å resolution model are given in Table 1. The model has three residues in the disallowed regions of the Ramachandran plot and 81.9% of residues in the most favored regions as defined by PROCHECK (Laskowski et al., 1993). The structure of p110β has residues 1-12, 228-234, 299-319, 402-431, 514-524 and 1058-1064 disordered. The regulatory p85β structure has residues 423-440, 501-507, 576-607 and 715-722 disordered. Figures depicting the structure were prepared with the program PYMOL (Schrödinger).

**Preparation of Liposomes**

Liposomes containing a defined mixture of brain lipids (we refer to these liposomes as “brain lipids”) contained 5% brain phosphatidylinositol-4,5-bisphosphate (Avanti 840046), 45% brain phosphatidylethanolamine (Avanti 830022), 15% brain phosphatidylcholine (Avanti 840053), 20% brain phosphatidylserine (Sigma P6641), 5% sphingomyelin (Sigma S0756) and 10% cholesterol (Avanti 700000). They were prepared in 2 mg batches by adding the lipid solutions into a 4 ml glass vial containing 130 µl chloroform/methanol (3:1) solution. The lipid solution was then dried to a film using an argon stream and desiccated under vacuum for 30 min. Lipids were then resuspended in 1 ml of a solution containing 20 mM Tris, 100 mM KCl, 1 mM EGTA, at a concentration of 2 mg/ml. The lipid suspension was sonicated in the glass vial with a bath sonicator for 5 min, before transferring it to a 1.5 ml Eppendorf tube and sonicating it for another 10 min. Subsequently, the liposomes were subjected to 10 cycles of freezing in liquid nitrogen followed by thawing at 42 °C for 2 min. Finally, the preparation was extruded with an Avanti Mini-Extruder 10 times through a 0.1 µm polycarbonate membrane (Nucleopore).

**In Vitro Kinase Activity Assay**

**Transcreener assay of ADP production**

The lipid kinase activity was determined using the Transcreener ADP2 FP Assay (BellBrook Labs, Madison, WI) according to manufacturer's specifications. This non-radioactive assay measures activity by quantitating the amount of ADP formed. The ADP formed by PI3K competes with fluorescent ADP tracer for binding to an anti-ADP antibody, thereby decreasing fluorescence polarization. Because of the competitive nature of this assay, the change in fluorescence polarization signal is not a linear function of enzyme activity, and a convenient measure of enzyme activity is the EC50, or the concentration of enzyme necessary to give half-maximal inhibition of labeled ADP binding to the antibody, for a given concentration of substrate and a fixed time of reaction at initial rate. While activity of two different enzyme constructs can be compared at a fixed concentration of protein and a fixed concentration of substrate, the assay is most reliable when about 2%-10% of the ATP is converted to substrate. This means that when comparing the most active constructs with the least active, the dynamic range is so large that these limits are quickly exceeded. Consequently, the EC50 provides a convenient way to compare the activities of various constructs. Briefly, reactions were performed in 10 μl volume in 384-well black plates (Corning 3676) and they contained final 100 μM ATP, 75 μM lipids and a series of protein concentration varying from 0.001 nM to 2.5 μM. Serial dilutions of protein heterodimers were prepared in buffer F (50 mM Hepes pH 7.5, 100 mM NaCl, 0.03% CHAPS, 4 mM DTT) in 5 μl volume. To initiate the reaction, 5 μl of a solution containing 200 μM ATP and 150 μM lipids in buffer G (50 mM Hepes pH 7.5, 100 mM NaCl, 0.03% CHAPS, 6 mM MgCl2, 2 mM EGTA) was added to each well. To compare the kinase activity in the presence of liposomes and soluble lipid substrate, the same reaction was carried out in the presence of either the lipid vesicles containing final 75 μM 1,2-dioctanoyl-sn-glycero-3-phosphatidylinositol 4,5-bisphosphate/1.425 mM 1-palmytoyl-2-oleoyl-sn-glycero-3-[phospho-L-serine] (diC8-$PIP_2$/POPS) (Invitrogen PV5100) or final 75 μM soluble diC8-$PIP_2$) (Echelon P-4508). The final concentration of ATP was 100 μM and protein concentration was varied from 0.001 nM to 2.5 μM. In single-point assays, protein concentration was fixed at 1 nM when using diC8-$PIP_2$/POPS, at 10 nM when using diC8-$PIP_2$ and 35 nM in the absence of lipid substrate (Figure 1D) or 75 nM (Figure S3B). The reaction was carried out for 1 h at room temperature.

To determine the effect of receptor tyrosine kinases (RTK) on PI3K activity, the assays were performed in absence or presence of 10 µM pY peptides:

1. Hs-c-kit-pY721+7: TNE(pY)MDMKPGV (residues 718-728)
2. Hs-c-kit-pY721+4: TNE(pY)MDMK (residues 718-725)
3. Hs-PDGFR-pY740+7: DGG(pY)MDMSKDE (residues 737-747)
4. Hs-PDGFR-pY740+4: DGG(pY)MDMS (residues 737-744)
5. Hs-PDGFR-pY751+7: SVD(pY)VPMLDMK (residues 748-758)
6. Hs-PDGFR-pY751+4: SVD(pY)VPML (residues 748-755)
7. Mm-PDGFR (pY2): ESDGG(pY)MDMSKDESID(pY)VPMLDMKGDIKYADIE (residues 735-767)
8. Hs-PDGFR-pY740+5: DGG(pY)MDMSK (residues 737-745)
9. Hs-PDGFR-pY740+6: DGG(pY)MDMSKD (residues 737-746)
10. Hs-PDGFR-pY740+5 position 5 mutated to P: DGG(pY)MDMSP
11. Hs-PDGFR-pY740+5 position 5 mutated to A: DGG(pY)MDMSA
12. Hs-PDGFR-pY740+5 position 5 mutated to S: DGG(pY)MDMSS
13. Hs-PDGFR-pY740+5 position 5 mutated to Y: DGG(pY)MDMSY
14. Hs-PDGFR-pY740+5 position 5 mutated to R: DGG(pY)MDMSR
15. Hs-PDGFR-pY740+5 position 5 mutated to E: DGG(pY)MDMSE
16. Hs-PDGFR-pY740+5 position 5 mutated to L: DGG(pY)MDMSL

All phosphopeptides were synthesized by Cambridge Peptides (http://www.cambridgepeptides.com).

The reactions were stopped by addition of 10 µl of stop/detection buffer (1X Stop & Detect Buffer B, 4 nM ADP Alexa633 Tracer, 109 µg/ml $ADP^2$ antibody), followed by 1 h equilibration. Fluorescence polarization was measured on a PHERAStar plus HTS microplate reader (BMG Labtech), using a fluorescence polarization module with excitation centered at 633 nm and emission at 650 nm. The data were fit in Prism (GraphPad) using a three-parameter exponential decay model.

**[$\gamma^{32}$P]-ATP assays of $PIP_3$ production**

In order to directly measure the $PIP_3$ production by the enzyme, we used the nitrocellulose-binding assay (Knight et al., 2007). Basically, 15 µl reactions having 0.1 µCi/µl [γ32P]-ATP, 100 µM ATP, 1 mg/ml lipid and 5 nM PI3K in a buffer containing 50 mM Hepes pH 7.5, 100 mM NaCl, 3 mM MgCl2 and 1 mM EGTA were incubated for 60 min at room temperature. A 2 µl aliquot of each reaction was then spotted onto a nitrocellulose filter. After spotting, the membrane was air-dried for 5 min and washed in 200 ml wash solution (1M NaCl, 1% phosphoric acid) for 30 sec. This was followed by five more washes with 200 ml wash buffer for 5 min each. The membrane was air-dried for 1 h, and then wrapped in a plastic wrap and exposed to a phosphoimager plate (Molecular Dynamics). The plate was exposed for 5 min and scanned by a Typhoon Scanner (GE Healthcare).

**Mass Spectrometry Assays of $PIP_3$ Production**

The $PIP_3$ lipids produced in PI3K *in vitro* assays as described above were also measured by a mass-spectrometry based assay (Clark et al.).

**Cell Culture, Transfection and Cell Lysis**

Phoenix cells (human embryonic kidney 293T-derived cells, Orbigen) were used to examine PKB phosphorylation levels in cells over-expressing wild-type or mutated p110β/p85α complexes. Cells were cultured at 37 °C, 5% $CO_2$ in RPMI-1640 GlutaMAX (Invitrogen) complemented with 10% heat inactivated foetal bovine serum, 100 U/ml Penicillin, 100 µg/ml Streptomycin. Transfection was done in 6-well plates using GeneJuice transfection reagent (Novagen) as described by the manufacturer. DNA (2 µg total) was used for each transfection, always using equal amounts of the catalytic and regulatory subunit DNA when co-transfected. Media was replaced with serum-free media 24 h following transfection and cells were grown for another 18 h. Cells were placed on ice, washed with ice-cold PBS and suspended in 150 µl lysis buffer (20 mM Tris-HCl pH 7.4, 150 mM sodium chloride, 1 mM EDTA, 1 mM EGTA, 1% Nonidet P40 (BDH laboratories), 2.5 mM sodium pyrophosphate, 10 mM sodium fluoride, 0.25% sodium deoxycholate, 1 mM AEBSF (Melford), 10 mM sodium orthovanadate and 1X protease inhibitors mixture (Roche)). Cells were then sonicated for 2 min, centrifuged 5 min at 13000 rpm and the supernatants were analyzed by Western blotting.

**Western Blot**

Protein levels in cell lysates were measured using the Bradford reagent and adjusted to 0.8 mg/ml with SDS sample buffer. After boiling for 30 min, 10 µl samples were run on a 4-12% Bis-Tris gel (Invitrogen) and wet-transferred to PVDF membrane. After transfer, membranes were blocked in 3% BSA in TBST (15 mM Tris pH 7.6, 150 mM NaCl, 0.1% Tween-20) for 1 h. Primary antibodies were incubated over-night at 4°C. Mouse antibodies recognizing PKB and phospho-PKB (Ser473) (Cell Signaling Technology, #2920 and #4051, 1:1000 dilution), myc (Santa Cruz, sc-40, 1:1000), FLAG (Sigma, F1804, 1:6000), actin (Boster Biological Technology, AC-40, 1:1000) and rabbit antibody recognizing phospho-PKB (Thr308) (Cell Signaling, #2965, 1:1000) were used as primary antibodies. Horseradish peroxidase (HRP)-conjugated anti-mouse (Santa Cruz, sc-2005, 1:7500) and anti-rabbit (Cell Signaling, #7074, 1:5000) antibodies were employed as secondary antibodies. Quantification of western blots was performed using GeneTools software (Syngene) and data analyzed in GraphPad Prism.

**Phospholipids Extraction**

To get a more direct measurement of PI3K activity in cells, we quantified total $PIP_3$ levels. To extract phospholipids, 1 ml of ice-cold 1N HCl was added to cells in a 3.5 cm well. Cells were scraped, transferred to a 1.5 ml tube and centrifuged at 13000 rpm for 5 min at 4°C. Supernatant (SN) was discarded and the pellet was resuspended in 750 µl methanol-chloroform-1N HCl (484:242:23.55), followed by 170 µl water. Samples were vortexed and allowed to stand for 5 min at room temperature. Phases were then split by addition of 725 µl chloroform and 170 µl of 2M HCl. Samples were vortexed and centrifuged at 5000 rpm for 5min. Lower organic phases were collected and transferred to 1.5 ml tubes containing 708 µl fresh 'upper phase' (upper phase taken from of a chloroform-methanol-0.01N HCl (240:120:90) solution. Samples were vortexed, spun down and the lower phase was collected into clean 1.5 ml tubes to be used for $PIP_3$ and $PIP_2$ quantification by mass spectrometry assay (manuscript under review).

**Differential Scanning Fluorimetry Assays**

Differential scanning fluorimetry measurement was performed to test for conditions that would stabilize p110β/p85β heterodimers (Niesen et al., 2007). In 96-well white plates (ABgene AB-0700/w), 36 μl of solution was prepared in buffer E (20 mM Tris pH 7.5, 100 mM NaCl, 5 mM DTT), containing complexes of p110β with truncated constructs of p85β encompassing the iSH2 and either the nSH2, the cSH2 or both domains. Final protein concentration was 300 nM. The solution was complemented with a range of additives, such as 100 μM pY peptides, 20 mM salts and various 0.5 mM PI3K inhibitors. 4μl of 10-fold concentrated SYPRO Orange dye (Invitrogen S6651) was added to the solution and the plate was sealed with Optical Adhesive Film (Applied Biosystems, No 4311971). Using a DNA Engine Opticon 2 Real-Time Cycler (BioRad), the plate was heated from 20 °C to 80 °C using 0.3 °C increments of 3 seconds and the fluorescence was read every 0.3 °C (excitation 470 nm/ emission 570). Melting temperatures (Tm) for each condition were derived from melting curves.

**Binding Assays of Phosphopeptides to p110β/p85α-icSH2 Complex**

In order to verify that all phosphopeptides (wild-type and mutant) used in the activity assays bind to the enzyme, we carried out competition binding assays. Binding of a fluorescein-labeled phosphopeptide derived from human PDGFR (739-744) (Fluor-GpYMDMS) to the p110β/p85α-icSH2 was first performed to determine the Kd of the fluorescent peptide. Two-fold serial dilutions of the p110β/p85α-icSH2 complex were prepared in binding buffer (50 mM Tris pH7.5, 100 mM NaCl and 1 mM TCEP) with highest protein concentration of 86 nM. Dilutions of p110β/p85α-icSH2 complex (15 μl) were added to 5 μl of a solution containing 2 nM fluorescent PDGFR peptide in a 384 well plate. The plate was incubated at room temperature for 10 min and fluorescence polarization was measured on a PHERAStar plus HTS microplate reader (BMG Labtech), using a fluorescence polarization module with excitation centered at 485 nm and emission at 520 nm. The data were fit in Prism (GraphPad) using a single-site binding model (Y=Bmax*[S]/(Kd+[S]), where Y is the fraction bound, Bmax is the maximum specific binding, [S] is the concentration of the labeled peptide and Kd is the dissociation constant for the peptide).

Subsequently, competition assays were performed in 384 well plates using the fluorescent peptide and the p110β/p85α-icSH2 complex. Two-fold serial dilutions of unlabeled peptides

were prepared in binding buffer with highest peptide concentration being 200 nM. Solutions were prepared with 10 µl of the unlabeled peptide solution and 10 µl of a solution containing 2 nM fluorescent PDGFR peptide and 6 nM of p110β/p85α-icSH2 complex protein. The plate was incubated at room temperature for 10 min and fluorescent polarization was measured using PHERAStar. An IC50 was obtained in Prism (GraphPad) using a competitive binding model (Equation: One site-fit logIC50). The Ki for the unlabeled peptide was determined using the Cheng-Prusoff equation (Ki=IC50/(1+[L]/Kd), where [L] is the concentration of the labeled peptide and Kd is the dissociation constant for the labeled peptide).

**Supplemental References**


Barber, D. F., Alvarado-Kristensson, M., Gonzalez-Garcia, A., Pulido, R., and Carrera, A. C. (2006). PTEN regulation, a novel function for the p85 subunit of phosphoinositide 3-kinase. Sci STKE *2006*, pe49.

Berndt, A., Miller, S., Williams, O., Le, D. D., Houseman, B. T., Pacold, J. I., Gorrec, F., Hon, W. C., Ren, P., Liu, Y. et al. (2010). The p110 delta structure: mechanisms for selectivity and potency of new PI(3)K inhibitors. Nat Chem Biol *6*, 117-124.

Blanc, E., Roversi, P., Vonrhein, C., Flensburg, C., Lea, S. M., and Bricogne, G. (2004). Refinement of severely incomplete structures with maximum likelihood in BUSTER-TNT. Acta Crystallogr D Biol Crystallogr *60*, 2210-2221.

CCP4 (1994). The CCP4 suite: programs for protein crystallography. Acta Crystallogr D Biol Crystallogr *50*, 760-763.

Clark, J., Anderson, K. E., Juvin, V., Smith, T. S., Karpe, F., Wakelam, M., Stephens, L., and Hawkins, P. Quantification of PtdInsP3 molecular species in cells and tissues by mass spectrometry. Nature Methods *In press*.

Emsley, P., and Cowtan, K. (2004). Coot: model-building tools for molecular graphics. Acta Crystallogr D Biol Crystallogr *60*, 2126-2132.

Foukas, L. C., Beeton, C. A., Jensen, J., Phillips, W. A., and Shepherd, P. R. (2004). Regulation of phosphoinositide 3-kinase by its intrinsic serine kinase activity in vivo. Mol Cell Biol *24*, 966-975.

Hale, B. G., Kerry, P. S., Jackson, D., Precious, B. L., Gray, A., Killip, M. J., Randall, R. E., and Russell, R. J. (2010). Structural insights into phosphoinositide 3-kinase activation by the influenza A virus NS1 protein. Proc Natl Acad Sci U S A *107*, 1954-1959.

He, J., de la Monte, S., and Wands, J. R. (2010). The p85beta regulatory subunit of PI3K serves as a substrate for PTEN protein phosphatase activity during insulin mediated signaling. Biochem Biophys Res Commun *397*, 513-519.

Knight, Z. A., Feldman, M. E., Balla, A., Balla, T., and Shokat, K. M. (2007). A membrane capture assay for lipid kinase activity. Nat Protoc *2*, 2459-2466.

Laskowski, R. A., MacArthur, M. W., Moss, D. S., and Thornton, J. M. (1993). PROCHECK: a program to check the stereochemical quality of protein structures. Journal of Applied Crystallography *26*, 283-291.

Leslie, A. G. (2006). The integration of macromolecular diffraction data. Acta Crystallogr D Biol Crystallogr *62*, 48-57.

McCoy, A. J. (2007). Solving structures of protein complexes by molecular replacement with Phaser. Acta Crystallogr D Biol Crystallogr *63*, 32-41.

Murshudov, G. N., Vagin, A. A., and Dodson, E. J. (1997). Refinement of macromolecular structures by the maximum-likelihood method. Acta Crystallogr D Biol Crystallogr *53*, 240-255.

Niesen, F. H., Berglund, H., and Vedadi, M. (2007). The use of differential scanning fluorimetry to detect ligand interactions that promote protein stability. Nat Protoc *2*, 2212-2221.

Rabinovsky, R., Pochanard, P., McNear, C., Brachmann, S. M., Duke-Cohan, J. S., Garraway, L. A., and Sellers, W. R. (2009). p85 Associates with unphosphorylated PTEN and the PTEN-associated complex. Mol Cell Biol *29*, 5377-5388.

Thompson, J. D., Higgins, D. G., and Gibson, T. J. (1994). CLUSTAL W: improving the sensitivity of progressive multiple sequence alignment through sequence weighting, position-specific gap penalties and weight matrix choice. Nucleic Acids Res *22*, 4673-4680.
