# Supplementary figures and images for: Structure of Lipid Kinase p110β/p85β Elucidates an Unusual SH2-Domain-Mediated Inhibitory Mechanism
Source: Mol Cell. 2011 Mar 4;41(5):567–78. doi: 10.1016/j.molcel.2011.01.026 (PMC3670040; doi:10.1016/j.molcel.2011.01.026)

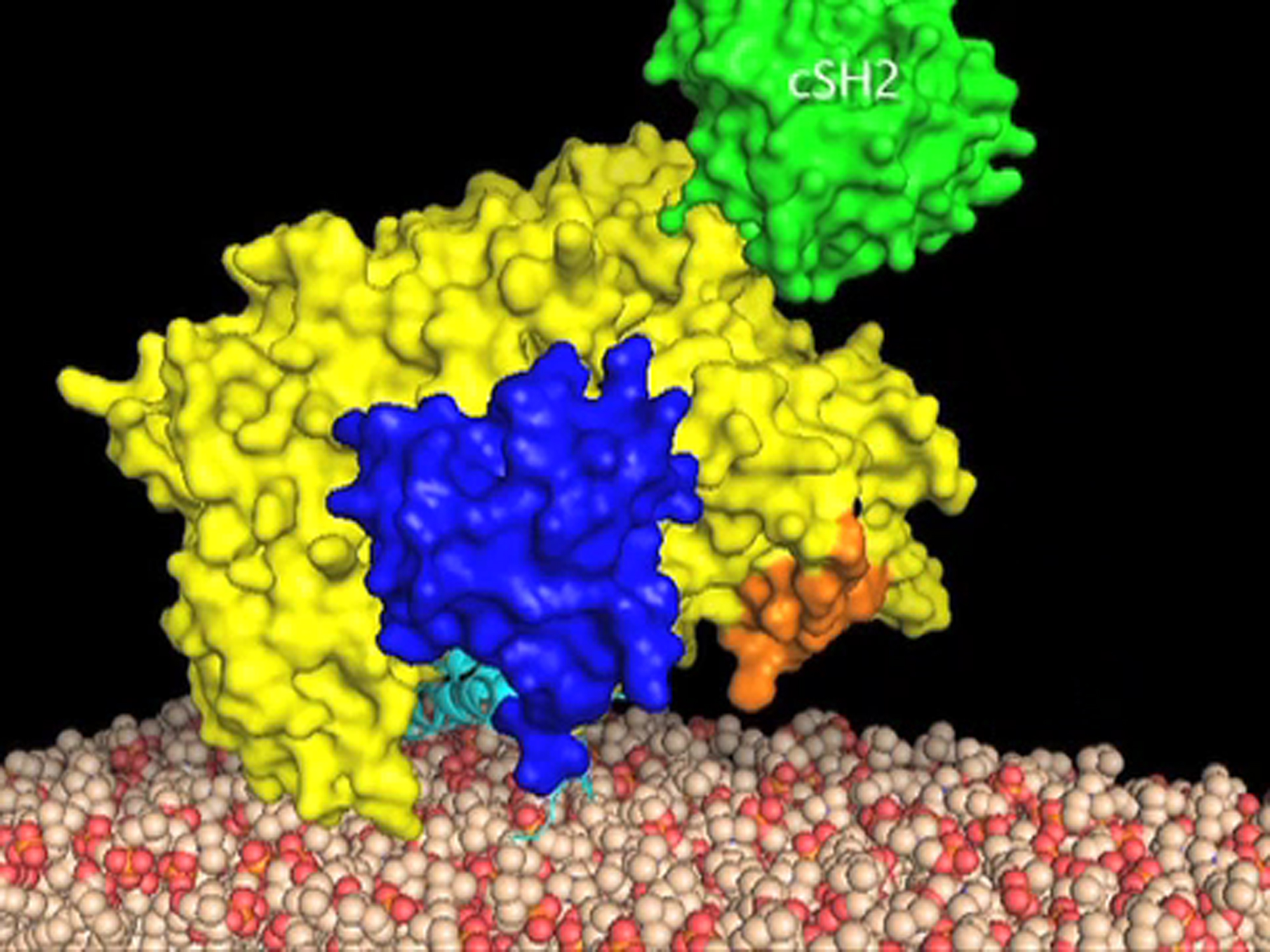

Supplement: Movie S1. Model of PI3K Beta Activation on Membranes [file mmc2.jpg]

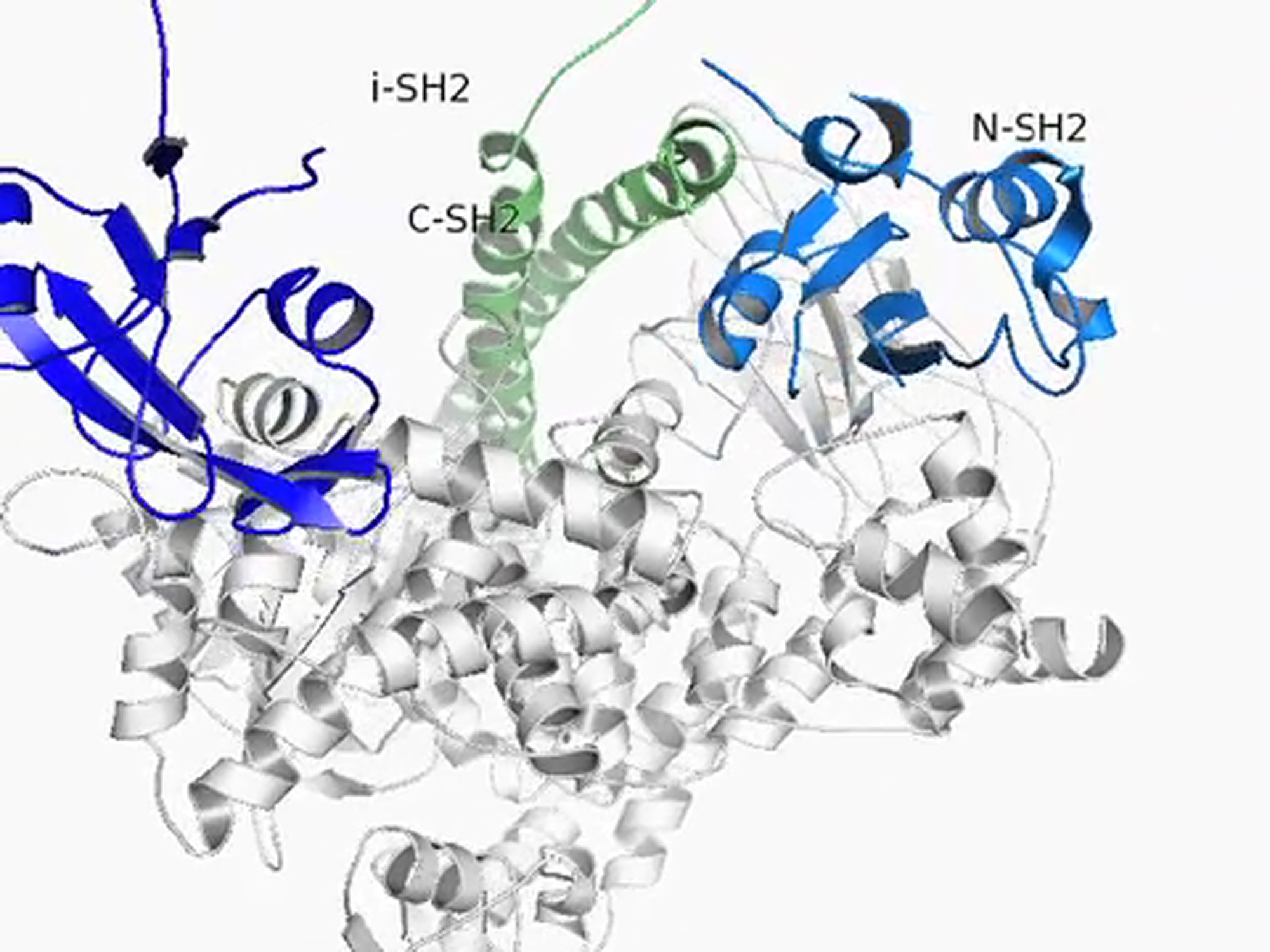

Supplement: Movie S2. Differential Mode of p110β Disinhibition by Phosphopeptide for the nSH2 and cSH2 — Movie S2 shows a model of nSH2 and cSH2 release from p110β by pY phosphopeptides (pY shown as yellow spheres). The pY binding site on the nSH2 is at the interface with p110β, whereas the pY binding site on the cSH2 is exposed. Phosphopeptides having at least five residues following the pY are necessary to break the contact of the cSH2 with p110β. [file mmc3.jpg]
